# Characterisation of the Structure and Oligomerisation of Islet Amyloid Polypeptides (IAPP): A Review of Molecular Dynamics Simulation Studies

**DOI:** 10.3390/molecules23092142

**Published:** 2018-08-25

**Authors:** Sandra J. Moore, Krushna Sonar, Prashant Bharadwaj, Evelyne Deplazes, Ricardo L. Mancera

**Affiliations:** 1 School of Pharmacy and Biomedical Sciences, Curtin Health Innovation Research Institute and Curtin Institute for Computation, Curtin University, GPO Box U1987, Perth, WA 6845, Australia; sandra.moore@student.curtin.edu.au (S.J.M.); k.sonar@postgrad.curtin.edu.au (K.S.); p.bharadwaj@ecu.edu.au (P.B.); evelyne.deplazes@curtin.edu.au (E.D.); 2 Centre of Excellence for Alzheimer’s Disease Research and Care, School of Medical and Health Sciences, 270 Joondalup Drive, Edith Cowan University, Joondalup, WA 6027, Australia

**Keywords:** amyloidogenesis, protein aggregation, molecular simulation

## Abstract

Human islet amyloid polypeptide (hIAPP) is a naturally occurring, intrinsically disordered protein whose abnormal aggregation into amyloid fibrils is a pathological feature in type 2 diabetes, and its cross-aggregation with amyloid beta has been linked to an increased risk of Alzheimer’s disease. The soluble, oligomeric forms of hIAPP are the most toxic to β-cells in the pancreas. However, the structure of these oligomeric forms is difficult to characterise because of their intrinsic disorder and their tendency to rapidly aggregate into insoluble fibrils. Experimental studies of hIAPP have generally used non-physiological conditions to prevent aggregation, and they have been unable to describe its soluble monomeric and oligomeric structure at physiological conditions. Molecular dynamics (MD) simulations offer an alternative for the detailed characterisation of the monomeric structure of hIAPP and its aggregation in aqueous solution. This paper reviews the knowledge that has been gained by the use of MD simulations, and its relationship to experimental data for both hIAPP and rat IAPP. In particular, the influence of the choice of force field and water models, the choice of initial structure, and the configurational sampling method used, are discussed in detail. Characterisation of the solution structure of hIAPP and its mechanism of oligomerisation is important to understanding its cellular toxicity and its role in disease states, and may ultimately offer new opportunities for therapeutic interventions.

## 1. Introduction

The incidence of type 2 diabetes (T2D) is rapidly increasing, and in 2015 it was the sixth leading cause of disability [1]. A World Health Organisation publication in 1980 reported that 108 million people worldwide were living with T2D. This number increased to 422 million in 2015, and it is expected to increase to 642 million by 2040 [2]. T2D is characterised by elevated blood glucose levels and insulin resistance [3]. T2D is a chronic metabolic disease that arises due to a lack of functionality of β-cells in the pancreas. Several mechanisms are believed to be responsible, such as the loss of β-cell mass, β-cell exhaustion, and the cytotoxic effects of elevated glucose and lipid levels [4]. The cause of T2D is, however, not fully understood.

A key hormone in the development of T2D is human islet amyloid polypeptide (hIAPP), also known as amylin [3]. hIAPP is stored in the pancreatic β-cells along with insulin secretory granules and it plays a role in the endocrine system and glucose regulation by slowing gastric emptying, reducing gastric secretion and promoting satiety [5]. Studies to understand the role of hIAPP in T2D have revealed two pieces of evidence. First, autopsy of 90% T2D patients showed the presence of hIAPP amyloid fibrils [6], and the degree of amyloid deposition correlates with the severity of the disease [7]. Second, formation of hIAPP amyloid fibrils is associated with reduced β-cell mass in both diabetic human [8] and non-human primates [9]. In addition, autopsy studies of the pancreas have shown the presence of extracellular hIAPP amyloid deposits around β-cells [10], and amyloid hIAPP aggregates have been found to be toxic to β-cells in in vitro studies [11,12].

hIAPP is a 37-residue long intrinsically disordered protein (IDP), which means that it is conformationally flexible, lacking a well-defined secondary and tertiary structure [13]. Soluble monomers of hIAPP exhibit a tendency to aggregate into soluble oligomers, which then go on to form insoluble amyloid fibrils [14]. Recent experimental studies have shown that the pre-fibrillary, early-stage soluble oligomers of hIAPP are very toxic, and more so than more mature amyloid fibril structures [15,16]. However, due to the difficulty in controlling and preventing aggregation, there is limited information about the structure of the soluble forms of hIAPP. The fibril structure of hIAPP, however, has been characterised experimentally, as described further below [10]. Further studies are thus needed to understand the structure and aggregation pathways of hIAPP in solution, which are likely to be important for the development of T2D. Molecular computational methods can be a complementary approach to study protein structure and interactions at the molecular level. Molecular dynamics (MD) simulations can provide an atomistic-level understanding of the structure and interactions of proteins that can be used in support of experimental data, with predictions that may be tested and validated under physiological conditions.

Characterisation of the solution structure of hIAPP in its monomeric form and its mechanism of oligomerisation is a key step in the understanding of its cellular toxicity and its role in disease. This could be instrumental for the prevention of the aggregation of hIAPP into toxic soluble oligomers and insoluble fibrils, and may offer a new therapeutic approach to treating or preventing T2D [17]. Other diseases may also benefit from this understanding. For example, the interaction of hIAPP with amyloid beta (Aβ) due to their accumulation in the brain may exacerbate neurodegeneration, and could be responsible for an increased incidence of Alzheimer’s disease (AD) in T2D patients [18,19,20]. Aβ is also an amyloidogenic protein whose soluble oligomers and insoluble fibrils are associated with AD [21]. The interaction between these two proteins has been seen to accelerate the development of AD. However, the interaction and possible cross-aggregation of these two proteins is poorly understood due to the limited structural knowledge of their soluble hetero-oligomeric forms.

## 2. Experimental Studies of the Structure of hIAPP

**Figure 1:**
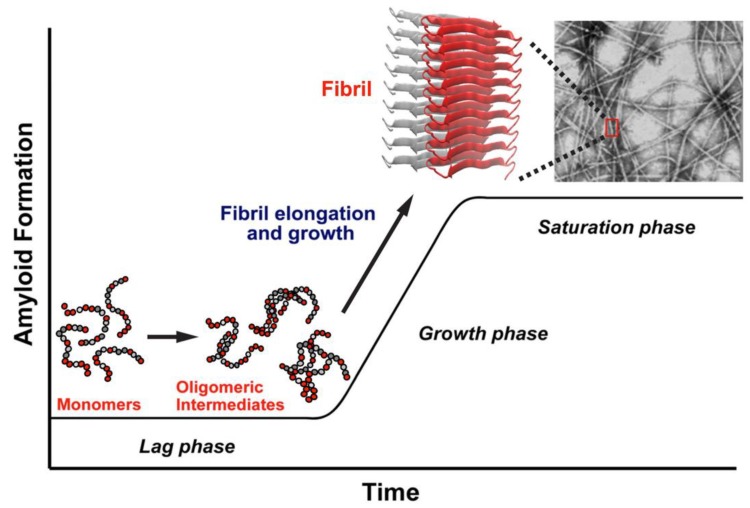
Schematic diagram of the kinetics of aggregation of human islet amyloid polypeptide (hIAPP). The early stages of aggregation (lag phase) involves the formation of disordered oligomers, followed by a rapid growth phase that leads to the formation of ordered fibrils, ending with a saturation phase when the fibrils have fully matured. Adapted from Abedini et al. [22]. Licensed under a Creative Commons Attribution (CC BY) license.

Figure 1 provides a simplified representation of the likely aggregation pathway of hIAPP. The highly unstructured monomers, dimers, and oligomers are soluble in solution, but at increasing concentrations, the oligomers aggregate and mature into insoluble fibrils. The soluble oligomers and insoluble fibrils are known to be toxic and are associated with β-cell death, leading to a decrease in insulin production and, ultimately, T2D [17].

The amyloidogenic process of aggregation of hIAPP is initiated by the interaction of one monomer with another one to form a dimer, after which the progressive interaction with more monomers leads to the formation of higher-order aggregates [23]. This aggregation begins with the “seeding” of one protein molecule with another, requiring a structurally disordered monomer to acquire a particular conformation that facilitates the interaction with another monomer. The process of aggregation progresses in time toward the formation of higher order soluble oligomeric and insoluble fibrillar states.

Sequence analysis followed by experimental testing have shown that hIAPP has a ‘core mutation region’ comprising residues 20–29 (Table 1, highlighted in blue) and which is part of the loop region of the β-strands in the fibrillar structure [24], as described in more detail below. This region is thought to be the amyloidogenic region of hIAPP, as it has been shown to be the smallest unit of hIAPP that aggregates, and substitutions within this region can prevent or slow the aggregation process [25,26]. A short peptide encompassing residues 20–29 of hIAPP has been shown experimentally to form an anti-parallel β-sheet conformation, as observed in the full length protein [25,27], making this peptide fibrillogenic. Another study used secondary structure prediction methods [28] to define a region in hIAPP that has a tendency to form β-sheet conformation, thus conferring amyloidogenic properties. 

**Figure 2:**
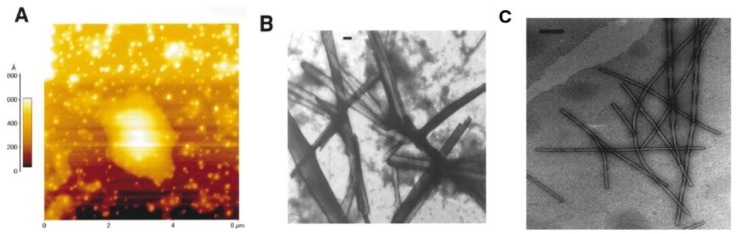
Atomic force (AFM) and electron microscope examination of aggregates of short sequences of hIAPP in its core mutation region. (**A**) AFM image24 (6 μm × 6 μm) of aged hIAPP_23–27_ (GAIL) in solution (10 mM). The vertical bar on the left-hand side of the figure indicates the heights of the measured objects. (**B**) Electron micrograph of insoluble aggregates of hIAPP_23–27_ (FGAIL) formed in an aged peptide solution. The apparent peptide concentration was 5.2 mg/mL in phosphate buffer (pH 7.4) with an incubation time of three days. The scale bar represents 200 nm. (**C**) Electron micrograph of insoluble aggregates of hIAPP_22–27_ (NFGAIL) formed in an aged peptide solution. The apparent peptide concentration was 6.4 mg/mL in phosphate buffer (pH 7.4) with an incubation time of three days. The scale bar represents 200 nm. Adapted from Tenidis et al. [30]. Copyright (2000) with permission from Elsevier.

In another study, a number of sequences in this core mutation region of hIAPP were investigated for their aggregation propensity: 22–27 (NFGAIL), 23–27 (FGAIL) and 24–27 (GAIL) [29]. Turbidity measurements, electron microscopy (EM), atomic force microscopy (AFM) and Congo red staining were used to determine that the 23–27 region (FGAIL) is the smallest sequence with the capacity to aggregate and form β-sheets. Rat islet amyloid polypeptide (rIAPP, Table 1), which does not aggregate or cause T2D in rats (see Section 2.3), lacks the same residues in this region. Aggregation of these peptides was conducted in highly concentrated aqueous solutions (3.7–9.3 mg/mL). As shown in Figure 2 [30], hIAPP_24–27_ (GAIL) formed round and punctuated aggregates, whilst hIAPP_23–27_ (FGAIL) and hIAPP_22–27_ (NFGAIL) aggregated into amyloid-like fibrils and fibril bundles. Although FGAIL and NFGAIL differ only by one residue, their fibril assemblies were completely different: FGAIL formed laterally arranged ribbon-like fibril bundles, while NFGAIL formed filaments coiled along each other, resulting in fibrils with axial helical periodicity. Furthermore, the kinetics of aggregation of these small peptides was determined to correspond to a nucleation-dependent polymerization mechanism. At similar molar concentration, the lag-time significantly decreased from 30 h for FGAIL, to 42 min for NFGAIL. These findings show the importance of Asn22, whose side chain might play an important role in intermolecular side-chain interactions that result in the association between two monomers. However there is some contention over the role of the core mutation region in the structural dynamics of hIAPP at various stages of its aggregation, as discussed later. A study of the aggregation kinetics of synthetic fibrils of hIAPP concluded that the core mutation region was essential for the formation of mature fibrils [31].

Helix-helix association has also been identified as being part of the mechanism of aggregation of hIAPP, in this case where aggregation is initiated by the interaction between a monomer in solution and another one present in a phospholipid bilayer [32]. A number of other amyloidogenic proteins have been observed to populate transiently helical structures, which are known to be stabilised by interactions with membranes. Helical intermediates bound to a membrane are believed to increase the local concentration of the aggregating proteins or, in particular, the core mutation region that initiates aggregation. It has been suggested that if membranes overstabilise helical structures, a decrease in aggregation would occur; however, this does not appear to be the case [33,34]. Nonetheless, this type of association has been observed in hIAPP in the presence of membranes, suggesting that lipids may be important in promoting aggregation in the complex, heterogeneous environment that is found in vivo [35,36]. 

Despite extensive characterisation of aggregation properties of the core mutation region, the study of the structure of the full length monomeric form of hIAPP has proven to be difficult due to two main reasons. First, hIAPP is an IDP and thus it lacks a well-defined structure. Characterisation of its structure with experimental methods such as X-ray diffraction (XRD), nuclear magnetic resonance (NMR), or circular dichroism (CD) spectroscopy have shown that the secondary and tertiary structures of hIAPP are not uniquely defined, suggesting that hIAPP transiently samples multiple conformations within a short period of time [37]. Second, as discussed above, hIAPP tends to aggregate rapidly at high concentration. In vivo hIAPP is soluble at concentrations of 1–4 mM, but it aggregates in vitro at a 1000-fold lower concentration [38]. Most of the above experimental methods require the protein under study to be present at relatively high concentrations [17]. Consequently, hIAPP in solution will have already aggregated when they are in the high concentrations needed for study, which prevents the ability to study the structure of its soluble forms. Characterisation of the structure of the monomer of hIAPP is, however, of uttermost importance to further the understanding of the mechanism of the formation of amyloid fibrils in T2D. The fibrillar form of hIAPP as the end point of aggregation has nonetheless been characterised, shedding light into the intra- and inter-molecular interactions present in this ordered, aggregate forms. 

### 2.1. Structure of Fibrils

**Figure 3:**
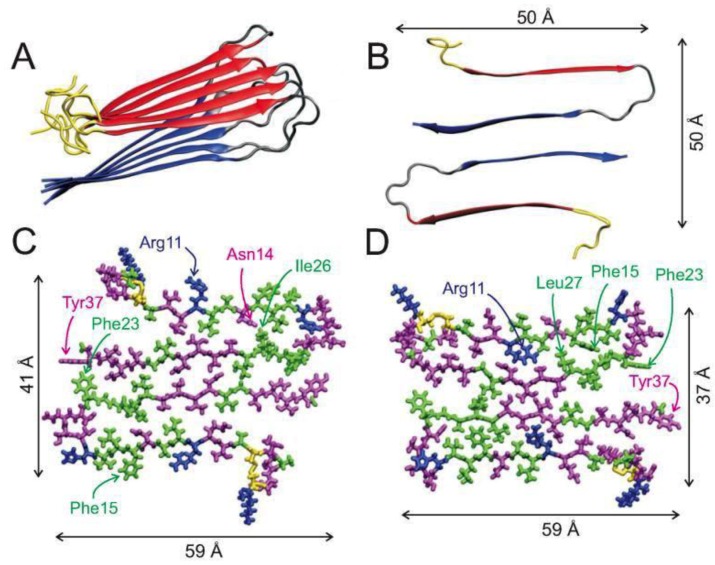
Structure of the fibrillary form of hIAPP: (**A**) Ribbon representation of the beta sheet conformation of hIAPP in fibrils determined by solid-state nuclear magnetic resonance (NMR) experiments; (**B**) Cross sectional view of two hIAPP monomers in the fibril form; (**C**,**D**) all atom representations of two possible models, with hydrophobic residues shown in green, polar in magenta, positively charged in blue, and the disulfide bond shown in yellow. Adapted from Luca et al. [40]. Copyright (2007) American Chemical Society.

CD spectroscopy, electron paramagnetic resonance (EPR), electron microscopy, two-dimensional infrared (2DIR) spectroscopy and solid-state NMR (ssNMR) have all shown that in its fibrillary form, hIAPP adopts a well-defined β-sheet conformation; however, the molecular structures of amylin fibrils have been difficult to obtain due to their lack of crystallinity and their insolubility [15,35,39,40,41,42,43,44]. X-ray and electron diffraction techniques have shown that the fibrils of hIAPP contain extended parallel β-strands that run perpendicular to the main axis [42,45,46,47]. Recent ssNMR studies of a single fibril structure characterised it as having a “cross-β-subunit consisting of stacked β-sheets formed from a repeating parallel U-shaped β-strand-loop-β-strand motif”, as shown in Figure 3A,B [40,43]. Measurement of NMR chemical shifts for C_α_ and C_β_ in hIAPP have demonstrated the presence of mostly positive secondary shifts towards C_β_, and negative shifts for CO and C_α_. This has suggested that the two β-strand segments comprise of residues 8–17 and 28–37, separated by a bend or loop comprising residues 18–27 (and encompassing the core mutation region, residues 20–29), as shown in Figure 3B [40]. Figure 3C,D show all atom representations of two potential models of hIAPP fibrils that differ mainly in the arrangement of side chains at residues 19–21, 24 and 25, which have been validated by 2DIR studies [43]. One of the models (Figure 3C) predicts that the β-sheet formation is stabilised by the side chains Gln10, Leu12, Asn14, and Leu16 making contact with each other, and with Arg11, Ala13, and Phe14 positioned on the exterior of the fibril. This model places residues Leu27, Ser29, and Asn31 at the interface of the fibril layers, whilst residues Leu26, Ser28, Thr30, and Val32 are placed on the interior of each layer. The second proposed model, shown in Figure 3D, differs to that in Figure 3C by having the β-sheet formed by contacts between residues Ala11, Ala13, and Phe15 and residues Gln10, Leu12, and Asn14a, with Leu16 being placed on the exterior of the fibril. It also shows residues Leu26, Ser28, Thr30, and Val32 at the interface, and residues Leu27, Ser29, and Asn31 on the interior of the fibril layers [40].

### 2.2. Structure of the Monomer

The full sequence of hIAPP is shown in Table 1, which also indicates that it possesses an amidated C-terminus and a (tight) disulfide bond between residues 2 and 7 [14,48,49]. The presence of the disulfide bond has been shown to be important in the stabilization, oligomerization, and amyloidogenicity in other amyloid proteins [50]. 

**Figure 4:**
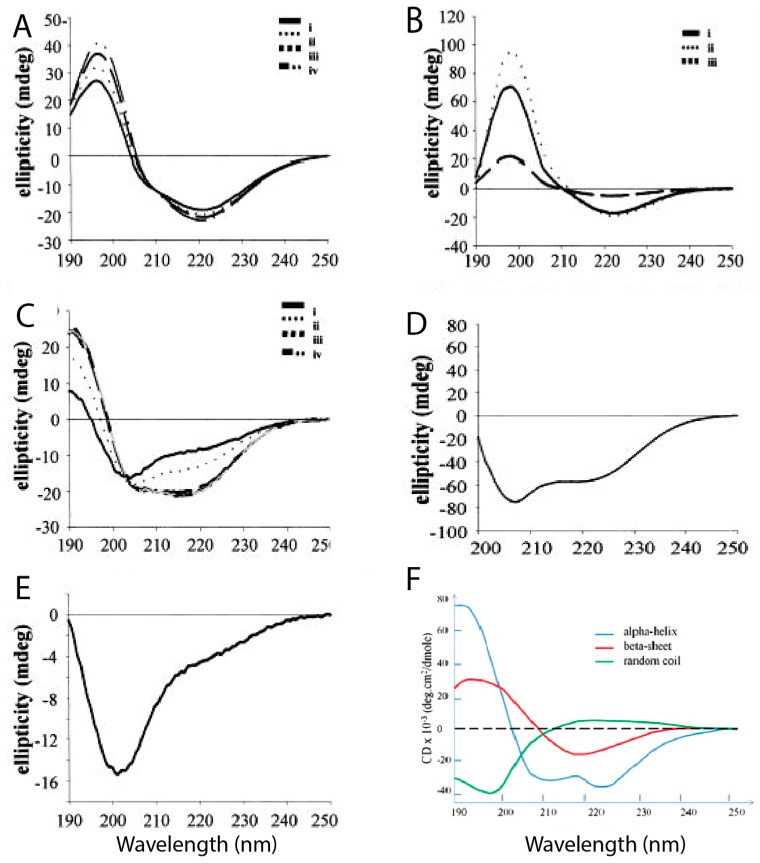
Circular dichroism (CD) spectra of human and rat IAPP in different preparations. (**A**) hIAPP in water at 0, 2, 8, and 24 h shows both α-helical and β-sheet contents, with an increase in β-sheet content over time, as seen by the increase in the peak at 200 nm; (**B**) hIAPP in 60% TFE at 0 h, 6 h, and 7 days shows an increase in β-sheet content over time; (**C**) hIAPP in 1% HFIP at 0 h, 10 min, 30 min, and 3 h is less stable and gradually converts from α-helical to β-sheet with time; (**D**) hIAPP in 10% HFIP after seven days is largely α-helical, as revealed by the curve in the 210–250 nm range (**E**) rIAPP in water after seven days shows a mostly random coil conformation; (**F**) reference curve for the ellipticity of α-helix, β-sheet, and random coil over different wavelengths. Adapted from Higham et al. [39]. Copyright (2000) John Wiley and Sons.

A limited number of experimental studies aimed at describing the structure of hIAPP monomers have been performed, all under non-physiological conditions to avoid aggregation. A study by Higham et al. [39] using CD spectroscopy and transmission electron microscopy (TEM) used trifluoroethanol (TFE) and hexafluoro-2-isopropanol (HFIP) to prevent aggregation, also using hIAPP in aqueous solution for comparison. Figure 4 shows the different CD spectra of hIAPP and rIAPP when prepared in different conditions. Figure 4E reveals that rIAPP in solution exists mostly in a random coil conformation, even after a prolonged period of time. Figure 4A–C shows that hIAPP initially has predominantly random coils and α-helical structures, but it progressively acquires more β-sheet content over time, during which it is assumed that aggregation is occurring [39]. Ion mass spectroscopy studies have also assessed the secondary structure of hIAPP (+4 charge) at different pH values. At lower pH (4.0) it was evident that the extended conformations were energetically favourable, and as the pH increased (up to 8.5), the more compact structures became more favourable [51].

**Figure 5:**
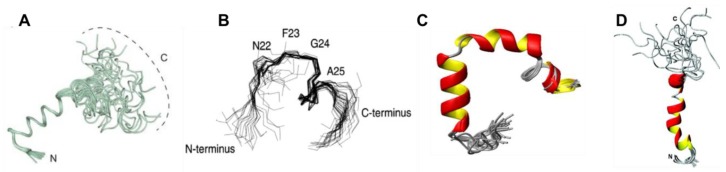
NMR structural ensembles: (**A**) hIAPP oxidised at pH 5.3 and at a temperature of 4 °C (PDB code 5MGQ). Adapted from Rodriguez Camargo et al. [52]. Copyright 2017 Rodriguez Carmargo et al. (**B**) Superposition of the hIAPP backbone Cα atoms for residues 20–29 (PDB code 1KUW). Adapted from Mascioni et al. [53] Copyright (2013) John Wiley and Sons. (**C**) hIAPP in a micelle environment (PDB code 2L86). Adapted from Nanga et al. [54] Copyright (2011) with permission from Elsevier. (**D**) rIAPP in a micelle environment (PDB code 2KJ7). Adapted from Nanga et al. [55]. Copyright (2009) American Chemical Society.

A recent study by Rodriguez Camargo et al. [52] demonstrated that redox conditions affect the aggregation of hIAPP, which was reduced and even halted by low, oxidising pH (5.3), low temperature (4 °C), and very low concentration (100 µM). CD spectroscopy measurements showed oxidised hIAPP to have α-helical and random coil secondary structures. NMR determinations allowed the generation of a conformational ensemble for this oxidised form of hIAPP, as illustrated in Figure 5A [52]. In particular, the N-terminal region of hIAPP (residues 5–19 and 20–22), which is known to sample β-sheet conformation, was also seen to transiently sample α-helical structures in solution. Removing the N-terminal disulfide bond (Cys2–Cys7) decreased the extent of α-helical content whilst it has also been shown to induce proteins to adopt β-sheet structure more easily [50].

Another approach to study the structure of hIAPP has been to characterise its structure whilst it is bound to a membrane-like environment. This approach is thought to be useful, as biological membranes are believed to play an important role in the misfolding and aggregation process of hIAPP. Cell membranes are thought to be a target for toxic oligomers, causing membrane disruption and toxicity [56]. Cell membranes can catalyse amyloidogenic aggregation, driving the conversion of α-helical structures into the β-sheet pleated structure found in fibrils [57]. Under optimal conditions, the interactions between hIAPP and cell membranes have been shown to increase aggregation and reduce the time for fibril formation from days to minutes [58]. Understanding the structure of hIAPP on a membrane is thus of considerable interest, and multiple studies have characterised the structure of hIAPP in a detergent micelle as a cell membrane-mimicking environment.

A recent study used two-dimensional solution NMR (2D-NMR) spectroscopy in sodium dodecyl sulphate (SDS) micelles to characterise specific regions of hIAPP, providing Hα chemical shift data [53]. The core mutation region (residues 20–29) was found to contain a bend at residues 22–25, and a β-turn at residues 26–28 (Figure 5B), which is consistent with this region being found in a bend or loop in the β-sheet fibrillar form. A solution NMR study by Nanga et al. [54] characterised micelle-bound hIAPP and observed it to exist predominantly in an α-helical conformation (Figure 5C). Electron paramagnetic resonance (EPR) studies have also been performed on hIAPP bound to a micelle. These studies again confirmed the presence of an α-helical region in the middle of the peptide (residues 9–22), with both terminal regions being observed to not adopt any detectable secondary structure. [59] This study was also in agreement with the above 2D-NMR study, and it found that the core mutation region (residues 20–29) is not in a stable conformation, and therefore it is more susceptible to misfolding. Studies with multiple other methods have come to similar conclusions about the α-helical nature of hIAPP when bound to a membrane, and it has thus been assumed that aggregation into fibrils is catalysed when the transiently sampled α-helical conformation is stabilised [54,60]. However, it is known that membrane environments can increase the α-helical propensity of hIAPP (and rIAPP) [61] so that the same conformational preferences cannot necessarily be assumed to exist for the monomeric form of hIAPP in solution.

### 2.3. Structure of Rat IAPP

**Figure 6:**
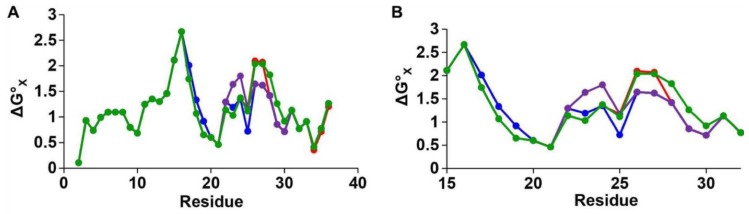
Hydrophobicity plots for hIAPP (red), r-IAPP (green), and a triple mutated version of hIAPP with substitutions at H18R, G14P, and I26P, which is non-toxic (TM-IAPP) (purple), and I26P-IAPP (blue), which is a substitution in hIAPP that is known to change aggregation propensity. Panel (**A**) describes the full sequence of IAPP, while panel (**B**) shows the key region of interest (residues 16 to 32). The vertical axis is residue solvation free energy as calculated from an octanol-to-water partition scale with mole-fraction units with glycine set to zero. Larger positive values indicate increased hydrophobicity. Adapted from Abedini et al. [22]. Licensed under a Creative Commons Attribution (CC BY) license.

Rat islet amyloid polypeptide (rIAPP) has been studied extensively for comparison with hIAPP. hIAPP and rIAPP differ by six residues (as indicated in Table 1); however, rIAPP does not aggregate, and T2D is not observed in rats [26,62]. Transgenic mice studies have confirmed that the amino acid differences between rIAPP and hIAPP are responsible for their distinct tendencies for aggregation: homozygous mice bred with hIAPP developed amyloid depositions, resulting in the spontaneous development of T2D [63]. Hydropathy plots (Figure 6) show the hydrophobic properties of each residue for both hIAPP and rIAPP, revealing that both proteins have very similar hydrophobic profiles [22]. Five of the six amino acid changes occur within the core mutation region of hIAPP. Three of these changes involve replacement with Pro in rIAPP, which are known as ‘secondary structure breakers’ [64]. Studies in α-helical proteins have shown that prolines decrease the propensity to form compact secondary structures [65]. Figure 5D shows the 2D-NMR structure of rIAPP in dodecylphosphocholine (DPC) micelles, which contains an α-helical region at residues 5–23 and a disordered C-terminus [55]. This figure illustrates a compact α-helical region for residues 5–17 and a disordered helix at residues 20–23. The presence of Pro 25, 28, and 29 (top of Figure 5D) leads to the absence of a compact secondary structure, and which differs to that of hIAPP (Figure 5C). It has been proposed that the lack of ability to form secondary structure in this region of rIAPP is the cause for its inability to aggregate [54,64,66].

NMR secondary structure chemical shift data has also provided evidence that the N-terminal half of rIAPP has upfield shifts that suggest the preference for α-helical confirmations. The Hα, Cα, and CO chemical shifts strongly indicate that residues 5–19 sample α-helical conformations. The tight disulfide bond between residues 2 and 7 is believed to limit the tendency for a well-defined secondary structure in the N-terminal region, but α-helical conformations were seen to be transiently sampled, which has also been observed with infrared (IR) spectroscopy and ion mass spectrometry [51,67,68].

## 3. Molecular Dynamics Simulation Studies of the Structure of rIAPP and hIAPP

MD simulations have been used to predict the structure of the monomeric forms of rIAPP and hIAPP in solution, as well as the self-aggregation of hIAPP. A wide variety of computational methods have been used to attempt to characterise the structure of the elusive IAPP monomer in solution, including different enhanced sampling methods, the use of implicit solvation methods, and both atomistic and coarse-grained force fields.

The first MD simulation study of full-length hIAPP identified three distinct, dominant conformational states in hIAPP: an extended β-hairpin structure, a compact α-helical structure, and a completely disordered random coil. This study used replica exchange MD (REMD) in the gas-phase and in implicit solvent to characterise both hIAPP and rIAPP. This study determined the β-sheet and α-helical propensities of hIAPP (36% and 6%, respectively), and rIAPP (7% and 36%, respectively) [51]. Residues 1–7 in both hIAPP and rIAPP did not exhibit β-sheet propensity, likely due to the conformational restriction caused by the disulfide bridge between residues 2 and 7. Both hIAPP and rIAPP exhibited α-helical propensity localized to the N-terminal half of the peptide (residues 1–25); however, this was more pronounced in rIAPP. By contrast, β-sheet propensity was predicted to be more pronounced for residues 8–37 in hIAPP. The core mutation region in rIAPP was predicted to have greater propensity to form turns and random coils than hIAPP, most likely due to the turn-prone prolines at positions 25, 28, and 29. These propensity differences suggest that the sequence differences between hIAPP and rIAPP have a global effect on the structure of these proteins, with hIAPP having an enhanced propensity to form β-sheet in the core mutation region. As the extended β-hairpin structural conformation is absent in both experimental and simulations studies of rIAPP, this structure was deemed to play an important role in the aggregation pathway of hIAPP. 

**Figure 7:**
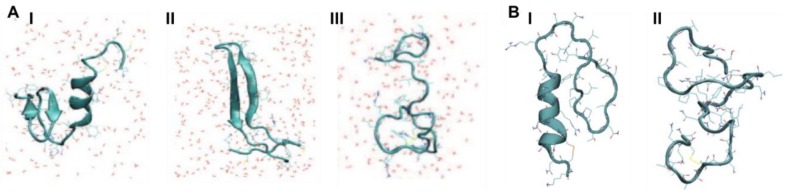
Representative predicted conformations of (**A**) hIAPP: (I) α-helical/coil, (II) anti-parallel β-sheet and (III) random coil conformation adapted from Reddy et al. [69]. (**B**) Representative conformations of rIAPP: (I) α-helical, and (II) random coil conformation. Reddy et al. [68]. Copyright (2010) with permission from Elsevier.

Two studies by Reddy et al. used REMD and replica exchange with umbrella sampling (REUS) to characterise the monomeric forms of both rIAPP and hIAPP. In both studies, an initial structure was constructed with α-helical content for each peptide, which was then described by an α-helical order parameter. Similarly to the above described studies, hIAPP was observed to frequent three stable conformations (Figure 7A). The first is a compact α-helical/coil structure with the helical segment comprised of residues 9–17, with a short anti-parallel β-sheet comprising residues 24–28 and 31–35 (Figure 7(AІ)) [68,69]. The second is an extended anti-parallel β-sheet structure with its turn region in residues 20–23 (Figure 7(AII)) [40]. The third is a completely unstructured conformation (random coil) (Figure 7(AШ)) [69]. Thermodynamic integration was used to determine the free energy differences between these three dominant conformations, revealing that the β-sheet conformation is more favourable than the random coil (−0.77 kJ/mol) and α-helical structure (−0.6 kJ/mol) [68,69]. By comparison, the transition from random coil to α-helical conformation in rIAPP is more favourable: −0.47 kJ/mol. The α-helical and β-hairpin conformations are stabilised enthalpically over the random coil conformation, with a larger enthalpic stabilisation for the α-helical conformation but with lower entropic stabilisation. This means that the α-helical conformation is even more stable at lower temperatures.

These studies also predicted that rIAPP has only two dominant conformations (Figure 7B) [68]. The first is a compact α-helical conformation comprising residues 7–17 (Figure 7(BІ)), and consistent with the NMR structure of rIAPP in a micellar environment (Figure 5D), and the second is an extended random coil conformation (Figure 7(BII)). At room temperature, the folded, α-helical structure is more dominant (55% vs. 45% for random coil), whereas at higher temperatures, the random-coil structure is more dominant. These structural predictions were validated by the calculation of experimental observables: the calculated α-carbon NMR chemical shifts were in good agreement with experiment, and the calculated IR spectrum in the amide I stretch regime was in fair agreement with the experimental line shape. It was concluded that the β-sheet conformation is a characteristic feature of amyloidogenic hIAPP [68,69].

Another key difference between hIAPP and rIAPP that has been predicted to play a role in aggregation is the presence of His18 in hIAPP. The β-hairpin structure present in hIAPP (+4 charge) but not in rIAPP, was shown to be stabilized, due to the repulsion between the positive charge of His18 and three positive charges at the N terminal region. When the β-hairpin structure is present, His18 and Arg11 are located at opposite ends of the β-strand, and the repulsion between these two residues likely stabilises the extended β-hairpin conformation. This is consistent with the increased rate of fibrillation that is observed at high pH in vitro, which may explain the pH-dependence of fibrillation in vivo. 

The conformational changes that initiate aggregation of hIAPP (and their equivalent in rIAPP) have also been assessed with MD simulations. The pathway from monomer to aggregate in hIAPP was predicted to involve the formation of contacts between the β-hairpin segment (residues 24–28 near the C-terminus) and the α-helical structure, leading to an overall loss of α-helicity, and unfolding of the peptide. The mutations to Pro in rIAPP (at residues 25, 28, and 29) prevent the formation of such a β-hairpin, thereby precluding the initiation of aggregation in the manner observed in hIAPP [68,69]. A MD bias-exchange metadynamics study tested the effect of these three Pro by creating a mutated version of hIAPP called pramlintide, which shares the three Pro substitutions with rIAPP. This study showed that the Pro substitutions reduce the stability and perturb β-sheet formation, thus reducing aggregation. The effect of each Pro substitution was investigated individually, and it was concluded that all three mutations are required for the disruption of β-sheet conformation and to reduce aggregation. These findings are thought to be important for the use of hIAPP-replacement treatments of T2D [70].

In order to understand the structural dynamics of monomeric hIAPP, Qiao et al. constructed a Markov state model (MSM) from extensive all-atom REMD simulations carried out using the AMBER99sb force field with TIP3P water model [71]. The starting structure in these simulations had α-helical conformation, as determined by sNMR in a micelle environment [72]. The conformational free energy landscape was computed, and multiple metastable states were identified. The majority of the conformations were in random coil, as observed experimentally [39]. The predicted radius of gyration ranged from 8.3 to 13.9 Å, which was comparable to the hydrodynamic radius of hIAPP (8.1 Å at 310 K) determined by diffusion NMR [73]. Mean first passage time (MFPT) analysis, which calculates the time taken from an initial to a final state, showed that the transitions between the metastable states were of time scales of several microseconds to milliseconds, indicating that the free energy of these states is well separated kinetically. The authors speculated that transitions between metastable states is slow, since it involves the disruption of existing contacts, the reorganisation of the conformation, and finally the formation of new contacts. Representative structures were analysed to identify aggregation prone conformations. β-hairpin segments encompassing 10 or more residues were found to be present along the protein length. In some representative structures, N-terminal residues in the range 11–18 were found to form an extended solvent-accessible hydrophobic surface that is exposed to the solvent, or an even larger hydrophobic cluster near the C-terminus formed by the residues 23–33. The presence of β-hairpin segments and extended hydrophobic surfaces make these metastable states aggregation prone, where the hydrophobic surface results in collapse of hIAPP through non-specific hydrophobic interactions. These interactions take place near the β-hairpin, which forms a topographically flat surface that acts as templates for other monomers.

**Figure 8:**
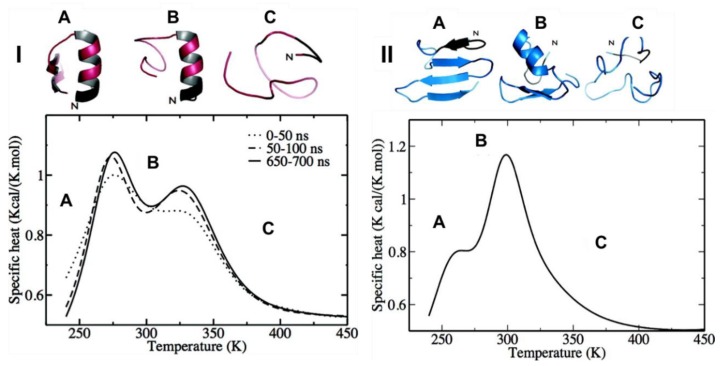
Specific heat capacity as a function of temperature at different time intervals for: (**I**) HT-REMD of hIAPP with representative structures for each peak, (**II**) HT-REMD of R-hIAPP with representative structures for each peak labelled A, B and C. Adapted from Laghaei et al. [74]. Copyright 2010 American Chemical Society.

Hamiltonian and temperature REMD (HT-REMD and T-REMD, respectively) simulations were performed to understand the role of the disulfide bond in the structure of the monomeric form of hIAPP. For WT-hIAPP (where the disulfide bond is present), heat capacity plots revealed the presence of two clear peaks (Figure 8(I)). At low temperatures (below 278 K) hIAPP transiently samples the α-helical conformation at residues 5–16, and a lesser visited β-hairpin with two β-strands at residues 22–27 and 30–36. The peak at 278 K suggests the α-helical structure (Figure 8(ІA)) remains intact whilst the β-hairpin of hIAPP is unfolding. The second peak at 322 K (Figure 8(ІB)) is indicative of complete unfolding of the secondary structures, with a smaller, short-lived α-helical structure still visible at times. At 300 K, the β-hairpin was seen only 5% of the time, whilst the α-helical structure remained stable. The same predictions were made for its reduced form (R-hIAPP; i.e., without its disulfide bridge). The heat capacity plots exhibited a small shoulder at 265 K (Figure 8(IIA)) and a significant peak at 299 K (Figure 8(IIB)). The shoulder at 265 K was representative of a four-part β-strand structure prior to the occurrence of an order-disorder transition of the N-terminal β-hairpin at 299 K to α-helical structure, before eventually reaching a completely unstructured random coil at 370 K (Figure 8(IIC)). At 300 K the β-hairpin at residues 22–27 and 30–35 was observed 30% of the time in the absence of the disulfide bridge. These findings suggest that while the disulfide bridge may play a significant role in stabilising the α-helical structure of hIAPP transiently sampled at residues 5–19, which could lead to aggregation [74]. In another MD simulation study of the reduced forms of both hIAPP and rIAPP, a reduction in α-helical content was observed when the disulfide bond was lost [75]. There are two reasons that have been suggested for the significant role of the disulfide bridge in hIAPP. Firstly, the disulfide bond in hIAPP stabilises α-helical conformation and is not involved in β-sheet structures. It is thought that the loss of the disulfide bridge decreases the stability of native structures and increases the propensity to form β-sheets and subsequent aggregation into oligomers and fibrils, which was evident in this study as more β-sheet conformations where seen in R-hIAPP. Secondly it is thought that the disulfide bridge prevents the interaction of highly amyloidogenic regions of hIAPP, and therefore limits aggregation [76]. Such roles for the disulfide bridge in hIAPP have also been described in other amyloid proteins and are thought to play an important role in aggregation [50].

### 3.1. The Choice of Force Field

There are multiple force fields that have been parameterised for proteins, usually with a specific choice of water model. Most force fields, however, are developed for globular proteins, which have well defined stable secondary and tertiary structures. This can cause overestimation of secondary structure content when it is used to characterise the structure of IDPs. The choice of force field is also heavily dependent on the protein being simulated, and the accuracy of a force field may vary from protein to protein. Consequently the choice of force field plays a critical role in the accuracy of the properties predicted by MD simulations [77,78]. This choice becomes more critical when considering IDPs, as there is limited experimental structural data, and so force field selection cannot be readily validated.

Protein force fields are continuously being developed and updated to correct for limitations or errors identified by comparison with experiments. Two improvements are particularly relevant to IDPs. The first is balancing the propensity of different secondary structures. This involves reducing the bias towards more structured conformations (secondary structures) by refining the backbone dihedral angles. The second improvement involves the balance between protein–water and protein–protein interactions. Force fields can overestimate hydrophobic burial of residues as IDPs rarely have hydrophobic cores [79]. This overestimation can also be attributed to the choice of the water model. Most force fields have undergone reparameterisation to further their ability to accurately describe the properties of IDPs. This has typically involved balancing the propensity of sampling secondary structures by refinement of backbone dihedral parameters and improving the balance of protein water and protein-protein interactions, often resulting in the introduction of pair-specific Lennard–Jones parameters. Best and Hummer have made efforts to improve the AMBER series of force fields with multiple reparameterisations, most notably the introduction of AMBERff99sb* and ff03* and their subsequent updates, which corrected the bias for underestimating and overestimating the helical contents of their predecessors. The CHARMM series of force fields have also undergone significant backbone refinements, with CHARMM36m being developed specifically for use with both folded and non-folded proteins. The OPLS force field has also had refinements to the backbone dihedrals and it has also has explored the use of residue-specific dihedral parameters [79]. 

**Figure 9:**
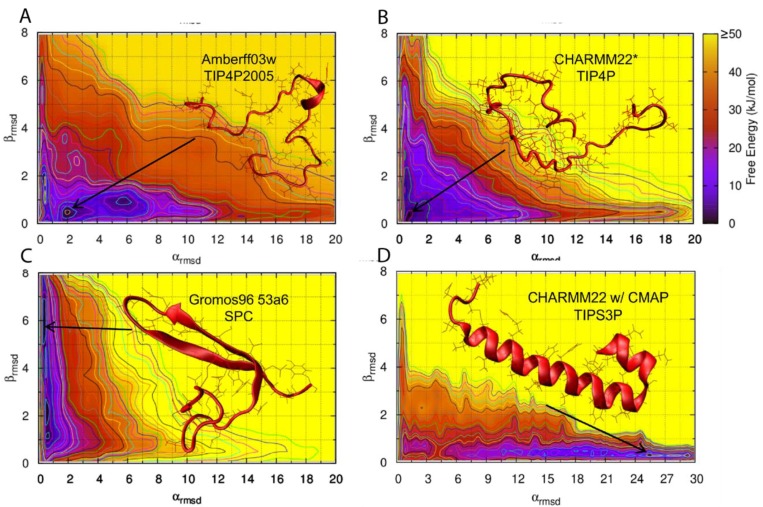
Conformational free energy landscape of hIAPP as a function of α-RMSD and β-RMSD for the AMBERff03w/TIP4P2005 (**A**), CHARMM22*/TIP4P (**B**), Gromos96 53a6/SPC (**C**), and CHARMM22/CMAP/TIPS3P (**D**) force fields. Adapted from Hoffmann et al. [62]. Copyright 2015 Hoffmann et al.

There have been a few studies that have tested the influence of force field choice on the predicted conformational landscape of hIAPPs/rIAPPs. However, due to a lack of experimental data, the choice of force field is not easily validated. A bias exchange metadynamics (BEMD) study of hIAPP and rIAPP tested six different force fields with various water models, namely Gromos96 53a6 with SPC [80], OPLS-AA/L with TIP4P [81], CHARMM22/CMAP with TIPS3P [82,83], CHARMM22* with TIPS3P and TIP4P [84], AMBERff99sb*-ILDN with TIP3P, and TIP4P [84,85,86,87], and AMBERff03w with TIP4P and TIP4P2005 [88]. Starting from α-helical structures obtained by solution NMR in a micellar environment [54,55], this study was conducted to understand how different force fields represent the structural ensemble of hIAPP and rIAPP [62]. The conformational landscape of hIAPP and rIAPP were generally observed to be similar, with differences arising in hIAPP readily adopting structures containing transient α-helices and β-strands. These transient conformations may enable dynamic aggregation pathways that eventually lead to the formation of amyloid fibrils. The secondary structure predictions from the conformational free energy landscape of rIAPP were compared to NMR Hα, Cα, and CO chemical shift data. AMBERff99sb*-ILDN with TIP4P, AMBERff03w with TIP4P2005, and CHARMM22* with TIP4P were the force fields that best represented the balance of secondary structures of both rIAPP and hIAPP in solution. The structure of hIAPP was predicted to be dominated by random coil, but with a much higher tendency to transiently sample α-helices and β-strands than rIAPP. Figure 9 shows the predicted conformational free energy landscapes and the most populated structure of the hIAPP monomer predicted by the two force fields deemed to be most accurate (AMBERff03w/TIP4P2005 and CHARMM22*/TIP4P), and the two force fields that show a clear bias towards a folded secondary structure (Gromos96 53a6/SPC and CHARMM22/CMAP/TIPS3P). It is evident there is variability between the location of the free energy minimum for all four free energy landscapes, with GROMOS96 53a7 with SPC over-predicting β-strand content and CHARMM22/CMAP over-predicting α-helical content in hIAPP [62].

**Figure 10:**
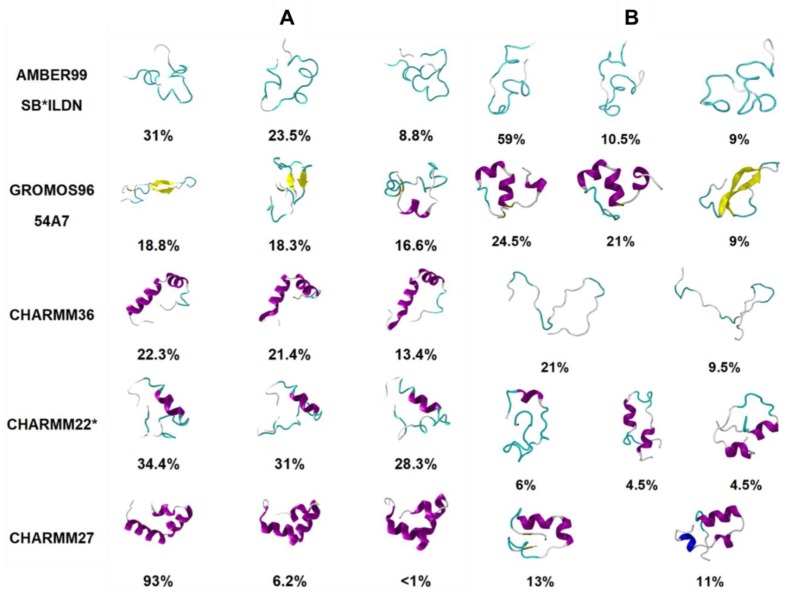
Conformational cluster analysis of REST2 simulations starting from (**A**) folded NMR structure and (**B**) disordered unfolded state of hIAPP. Top clusters for each simulation were selected to represent the most common conformations of the equilibrated trajectory. α-helix is represented in blue, β-sheet in yellow, random coil in white, and turn in cyan. Adapted from Peng et al. [89]. Copyright 2017 Peng et al.

Another study by Peng et al. [89] tested five different force fields with various water models (AMBER99SB*-ILDN with TIP3P [84,85,86,87], Gromos 54a7 with SPC [79], CHARMM36 with TIPS3P [90], CHARMM22* with TIP3P and TIPS3P [84], and CHARMM27 with TIP3P and TIPS3P [83]) for the prediction of the conformational free energy landscape of hIAPP. The study used both conventional (unbiased) MD (cMD) and replica exchange with solute tempering (REST2) methods. Two different starting structures were tested: random coil and the folded α-helical structure in SDS micelles obtained by solution NMR (PDB code 2L86) reported by Nanga et al. [54] This study, however, did not study rIAPP and, instead, made a comparison to NMR and IR experimental data of hIAPP [91]. Certain force fields like CHARMM27 and CHARMM36 were observed to be unable to explore conformations away from the starting α-helical structure, using both cMD and REST2 simulations, suggesting the possible influence of the choice of starting structure (see below). The CHARMM27 and CHARMM36 force fields demonstrated a clear bias towards α-helical structures, while Gromos 54a7 exhibited a bias towards β-sheet-dominant structures. This is evident in Figure 10, where the different dominant conformations predicted by each force field are shown. The clear prediction of a compact secondary structure in solution (e.g., CHARMM27 predicts hIAPP to be primarily α-helical in structure) is not characteristic of IDPs, nor has it been observed experimentally for hIAPP. These predictions were compared to IR and ion mass spectroscopy data available for the monomeric form of hIAPP, which showed that AMBER99SB*-ILDN with TIP3P, and CHARMM22* with TIP3P showed the best agreement with experiment, exhibiting a balance between disordered conformations and conformations with some α-helical content [89]. No comparison was made to predictions of structure for the monomeric form of rIAPP.

Both of the above studies aimed to identify the most appropriate force field by comparing the predictions of experimental observables to experimental and/or previous simulation data. CHARMM22* with TIP3P was found to be appropriate in both studies, but there is still large variability between the predictions of the other force fields. To directly compare BEMD and REST2 the same conditions are required to ensure that no other factors such as the starting structure, the water model, and/or the length of the simulation, influenced the conformations sampled. The large variability between methods and force fields makes conclusions about the structure of hIAPP and rIAPP limited, and more systematic and in-depth research is thus needed. 

### 3.2. Influence of the Starting Structure

The choice of starting structure in MD simulations of IDPs can play a critical role in the extent of conformational sampling, as mentioned above. Enhanced sampling methods aim to eliminate the influence of the starting structure, but this has not been extensively tested for hIAPP or rIAPP. There may be energy barriers between different states of hIAPP/rIAPP, which could make the choice of starting structure critical to enable the exploration of the entire conformational free energy landscape. At the same time, the enhanced sampling method chosen would need to be tested to demonstrate that the starting structure does not limit or bias the exploration of the energy landscape. 

Peng et al. [89] characterised the monomeric structure of hIAPP using two different starting structures (a random coil and the folded α-helical structure in SDS micelles obtained by solution NMR), as described above. The authors referred to the limitation of other studies that have not compared starting structures and the potential bias the simulation predictions could have towards a folded starting structure. Use of cMD simulations appeared to lead to an increase in α-helical propensity when the helical starting structure was used. Interestingly, this was also observed when enhanced sampling (with the REST2 method) was used (Figure 10). Figure 10 shows the influence of the starting structure with the CHARMM36 force field, clearly demonstrating that the helical starting structure biased the simulations. The authors concluded that enhanced sampling simulations, starting from a completely random coil, result in the most representative portrayal of the conformational free energy landscape of the hIAPP monomer [89].

### 3.3. The Choice of Enhanced Sampling Method

It appears that the choice of enhanced sampling method, used to characterise different conformations of hIAPP, may also play a critical role in the accuracy of the predicted conformational free energy landscape. cMD simulations have large limitations in sampling, especially for IDPs, which do not remain in a stable conformation but rather transiently sample many structures. The extensive and complex free energy landscapes of IDPs, and of hIAPP in particular, are unlikely to be sampled efficiently by cMD on feasible time scales, if at all. This can cause simulations to continually sample only a small portion of all potential conformations. To overcome this limitation, enhanced sampling methods have been developed that can bias the system to sample conformations that may not be energetically favourable in order to explore the entire free energy landscape in shorter time scales [92]. Most MD simulations reported so far aimed at characterising the monomeric structure of hIAPP have used enhanced sampling methods, but have not determined whether there is an optimum method, or whether most methods give similar results. To accurately sample the conformational free energy landscape of hIAPP, agreement between converged simulations using different enhanced sampling methods is necessary after accounting for all other factors.

Hoffmann et al. [62] used BEMD to simulate the conformational free energy landscape of the monomeric form of hIAPP. BEMD uses collective variables (CVs) to bias simulations into sampling different conformations. A history-dependent potential is created by adding positive (repulsive) Gaussian potentials, which are added to prevent sampling of conformations already visited. The free energy landscape (e.g., Figure 9) can be reconstructed after the simulation has been completed [93]. BEMD is a variant of the metadynamics (metaD) method whereby a larger number of collective variables are used. Hoffmann et al., however, only used two CVs, α-RMSD and β-RMSD, which measures the similarity to ideal structures of α-helix and β-sheets. It is evident from Figure 9 that different force fields predict different free energy landscapes, which is especially evident in CHARMM22/CMAP and Gromos96 53a7. This study reached the broad conclusion that hIAPP was predicted to be mostly in random coil conformations but also transiently sample both α-helical and β-strand structures, which is in agreement with earlier experimental and simulation studies.

Zerze et al. [94] compared different enhanced sampling methods when they characterised the structure of the monomeric form of hIAPP. BEMD was compared to temperature replica exchange MD (T-REMD); however, only one force field was tested (AMBERff03w with the TIP4P2005 water model). For the BEMD method, seven CVs were used: hydrophobic contacts coordination, α-RMSD for residues 8–22, α-RMSD for residues 22–36, parallel β-RMSD, anti-parallel β-RMSD, Cα-Cα contacts coordination, and ψ-dihedral angle correlation. The hydrophobic contact coordination and the Cα-Cα contacts coordination numbers were used to measure the compactness of the protein, and the ψ-dihedral angle correlation was used to measure local structures such as PPII. These additional CVs may not provide much additional information that could not be obtained from using only α- and β-RMSD. The T-REMD simulations were post-processed to extract the properties defined in the same way as the CVs, to compare the sampling performance of both methods. Predictions with both methods showed agreement for both free energy profiles and secondary structure analysis. BEMD was able to sample a larger region in the free energy surface, but it had a higher computational cost and a longer initial benchmarking process. Nonetheless, it was concluded that both methods are highly suitable for studying the secondary structural propensities of hIAPP. 

Peng et al. [89] characterised the structure of the monomer of hIAPP using REST2, whilst also comparing to cMD simulations. The REST2 method involves the use of multiple replicas with different Hamiltonians, whereby the interactions of a solute are modulated to increase sampling whilst lowering the number of replicas, making it computationally more efficient. [95] The study demonstrated the necessity for enhanced sampling methods as there was a large influence of the choice of starting structures (see Section 3.2 above), as well as evident differences between the five force fields used (Section 3.1). Simulations with the REST2 method also exhibited a bias when starting from an α-helical conformation. The authors found reasonable agreement between their REST2 simulations and the BEMD simulations performed by Hoffmann et al. [62] and Zerze et al. [94]. 

A study performed by Laghaei et al. [74] characterised the structure of hIAPP using two enhanced sampling methods with the coarse-grained OPEP force field in implicit solvent. This study compared T-REMD and Hamiltonian-REMD (HT-REMD), demonstrating that both methods arrived at the same predictions. Figure 8 shows the predicted heat capacity plots for hIAPP using both HT-REMD and T-REMD, and it is evident that both methods predicted very similar thermodynamic behaviour; however, HT-REMD was determined to reach convergence faster. Figure 8 shows the heat capacity plots, with two defined peaks in similar positions predicted by each method. As mentioned above, secondary structure predictions showed mostly random coil conformations, but with a relatively stable α-helical structure at residues 5–16 and a weaker β-sheet region spanning residues 22–26 and 30–35, which is consistent with predictions made by other simulations using different enhanced sampling methods.

It is clear that there is limited consensus between experimental and simulation data on the structural characteristics of the monomer of hIAPP in solution under physiological conditions. However, the structural characteristics of the monomeric form have been used in MD simulation studies to try to understand the process of aggregation of hIAPP. 

## 4. MD Simulations Studies of the Initial Stages of Aggregation of hIAPP

Aggregation or amyloidogenesis is the phenomenon that results in the formation of higher order aggregates and eventually fibrils by the interaction of monomeric peptides of an amyloid protein such as hIAPP. In order to understand this process, it is crucial to know what region of the 37-residue sequence is primarily responsible for the initial interaction between two monomers to form the smallest unit of aggregation, i.e., a dimer. Different mechanisms have been suggested to cause the aggregation of hIAPP, likely first involving changes to the secondary structure preferences of monomers in solution to conformations that facilitate interaction between monomers. Several MD simulation studies have been performed to try to understand this phenomenon.

**Figure 11:**
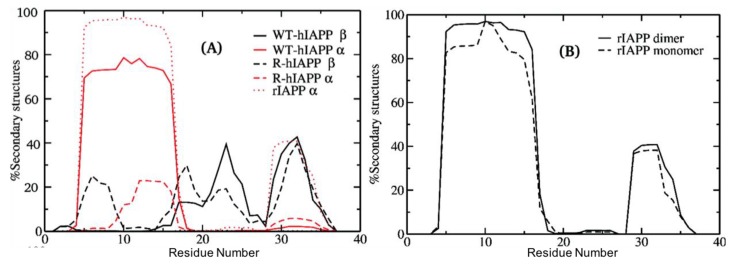
Differences in secondary structure content of monomers and dimers of hIAPP and rIAPP. (**A**) WT-hIAPP and R-hIAPP dimers; (**B**) rIAPP monomer and dimer. Adapted from Laghaei, et al. [96]. Copyright (2011) American Chemical Society.

The study by Langhaei et al. [96] using HT-REMD simulations also characterised dimers of wild-type (WT) hIAPP, reduced hIAPP (R-hIAPP), and rIAPP. The process of dimerization was simulated, starting from random conformations and allowing the monomers to sample their mutual configurations. Convergence was achieved when the entropy of the dimer was observed to be stable. In dimeric systems of WT-hIAPP, residues 5–16 exhibited a high tendency to form α-helixes (>75%), as shown in Figure 11, whilst β-sheet content was observed for residues 17–27 and 29–35 (up to 40%). Strong hydrophobic interactions were observed between the side chains of residues L12–F15 in one monomer, and L12–L16 in the other one, which may imply the presence of helix-helix association in dimers of WT-hIAPP. As described at the end of Section 3 and Section 3.3, these authors predicted that monomers have a similar tendency to exhibit α-helical content, but the tendency for β-sheet content was higher in the dimer for residues 17–27 and 29–35, and lower in monomers for residues 22–26 and 30–35. On other hand, in the dimer of R-hIAPP, the absence of the disulfide bond resulted in the destabilization of α-helical conformations; however, residues 5–9 were now predicted to exhibit β-sheet content (5–24%). In the C-terminal region, β-sheet content is predicted to be similar to that of WT-hIAPP. Dimerization was predicted in general to increase the propensity to form stable secondary structures in the dimer of WT-hIAPP with respect to the monomer. On the other hand, the combined α-helical and β-sheet content in the dimer of R-hIAPP was predicted to be 18%, which is significantly lower than that observed in the monomer (28%). Compared to WT-hIAPP, the inter-chain side chain-side chain atomic contact probability was observed to drastically decrease for residues 1–9 and was around 60% for the region 12–16 in the dimer of R-hIAPP. The monomer of rIAPP exhibited two regions with α-helical content: residues 5–16 (96%) and 29–34 (38%). The N-terminal region thus was observed to have conformational preferences that were very similar to those of hIAPP, and both the monomer and dimer of rIAPP were indeed predicted to have very similar secondary structure content (Figure 11B), comprising α-helical structure in the N-terminal region, with a distortion in the middle of the chain and with another smaller α-helix, without any β-sheet content, consistent with solution NMR determinations in a micelle environment [96]. Analysis of inter-chain interactions revealed that there are residues with high contact probabilities in rIAPP (L12–L16, F15–L16, L12–I26, L12–L27, L16–L27, I26–V32, and V32–L27). In contrast to WT-hIAPP, reduced interaction between the amyloidogenic regions (residues 22–27 and 30–37) was predicted.

Another cMD simulation study using the AMBER99sb force field with implicit solvation aimed to characterise the structure of the monomer and dimer of hIAPP, as extracted from pre-formed fibrils [40]. Hydrophobic collapse in the monomers was observed during the initial part of the simulation, leading to a more globular structure. Dimers were predicted to have higher stability, and they maintained their initial fibril-like structure, which may have been due to their buried hydrophobic residues contributing to the stabilization of the dimer [97]. 

**Figure 12:**
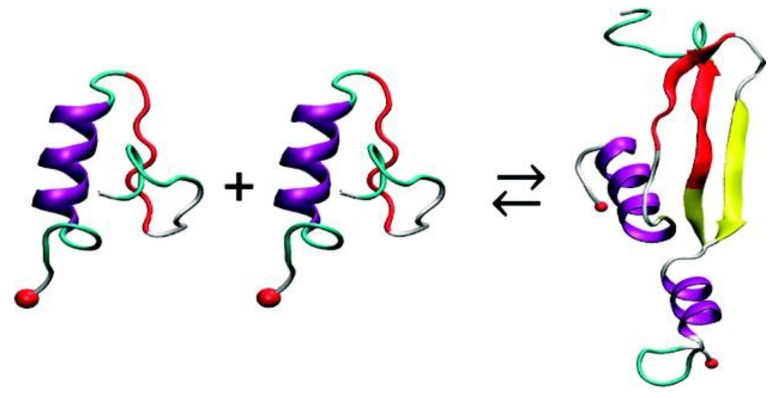
Dimerization of hIAPP with conversion of helix–coil to helix–β-strand structures (adapted from Dupuis et al. [98]. Copyright (2011) American Chemical Society.

A REMD simulation study using the Amberff96 force field in implicit solvent characterised monomers of hIAPP and rIAPP obtained by ion mobility mass spectrometry from a previous study [51]. The formation of homodimers was simulated by initially placing the monomers far apart. The interface of the homodimer of hIAPP comprised segments that formed β-strands (residues 11–18 and 23–32) as well as the core mutation region, but not the N-terminal region. The side-by-side assembly between the β-hairpins was observed to be the major contributor to the stability of the structure. These β-strands were observed to be similar to those observed experimentally in fibrils (residues 8–18 and 22–28). On the other hand, the monomers of rIAPP were not observed to form β-strands (due to their Pro residues), and hence they were observed to interact via random coil structures, although this was not energetically favourable compared to the interaction between monomers of hIAPP: the free energy of binding for monomers of rIAPP was predicted to be −38.3 kcal/mol compared to −59.5 kcal/mol for monomers of hIAPP. In the formation of the dimer of hIAPP, the initial α-helical and random coil conformations (residues 19–37) of the monomers were predicted to transition to α-helix and β-strand conformations, as shown in Figure 12 [98]. 

**Figure 13:**
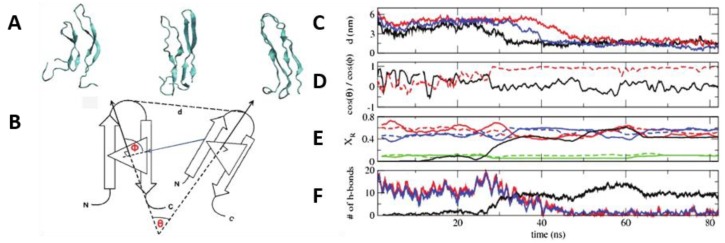
Characterisation of the formation of dimers of hIAPP. (**A**) Representative configurations along the dimerisation pathway of two human amylin peptides in their β-hairpin conformation. The time evolution of the process of dimerisation was characterised, taking the distance between monomers using the turn-forming Asn22 (**B**), the angles between orientation vectors ((**C**,**D**) with schematic representation (**B**)) (first, molecules at a distance of 5 nm, with a θ angle between the two peptides of 90° (**D**, shown in black); second, molecules at a distance of 6 nm, with a θ angle between them of 180° (shown in red)) in each monomer, secondary structure (**E**), the number of main chain intra-strand hydrogen bonds in the two monomers, and the number of main chain inter-strand hydrogen bonds (**F**). Adapted from Reddy et al. [69]. Copyright (2010) with permission from Elsevier.

In the study by Reddy et al. [69] discussed earlier, REMD simulations were also conducted to simulate the dimerization of hIAPP. In order to minimise sampling bias, multiple replicas of the system were used, whereby the monomers were placed at different distances and at different initial relative orientations. It was observed that, irrespective of their initial relative orientation, the monomers approached each other during the course of simulation (20–60 ns) and formed a dimer, during which intra-strand main chain hydrogen bonds were lost and inter-strand hydrogen bonds were made, stabilising the dimer (Figure 13) [69].

**Figure 14:**
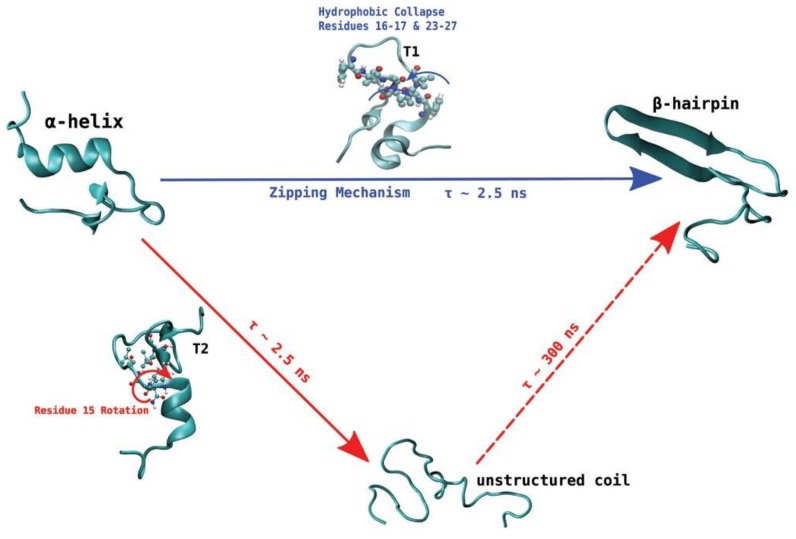
Proposed pathways of transition of hIAPP from an α-helical conformation to a β-hairpin. The zipping mechanism (in blue) involves the gradual loss in α-helical structure by the gain of β-hairpin characteristics. The second mechanism (in red) is a two-step transition process that involves the transformation of the α-helical state into an unstructured coil before eventually transitioning to β-hairpin. Adapted from Singh et al. [99] with the permission of AIP Publishing.

De Pablo and his collaborators proposed two different pathways that can initiate the process of aggregation of hIAPP. Unlike other amyloidogenic proteins, whose aggregation requires the initial complete unfolding into a random coil followed by gradual reorganisation into a β-sheet aggregate, the evidence discussed so far seems to indicate that hIAPP may retain some β-hairpin conformation during the initial stage of aggregation. Transition path sampling REMD simulations with the Gromos96 53a6 force field were used to predict the most likely mechanism of folding between the most dominant conformations in monomeric hIAPP [99]. In particular, the α-helical conformation was determined to undergo a transformation into the β-hairpin conformation through one of two mechanisms, as a likely precursor to aggregation. In the first one (Figure 14, shown in blue) misfolding proceeds via a zipping mechanism. This process is initiated by the hydrophobic collapse of residues 16–17 and 23–27, which causes the formation of a β-turn in the core mutation region. The second one (Figure 14, shown in red) involves misfolding through an unstructured coil intermediate, thought to be caused by rotation of Phe15 towards the interior of the peptide, which leads to the gradual loss of the transient α-helical conformation. Both mechanisms were predicted to exhibit similar free energy barriers with inter-conversion between the α-helical conformation and β-hairpin being an activated process, which could constitute the nucleation event for aggregation and subsequent fibrillation. The significance of the role of the hydrophobic collapse in initiating the process, however, was deemed to remain in doubt due to the use of implicit solvation in the simulations [99].

**Figure 15:**
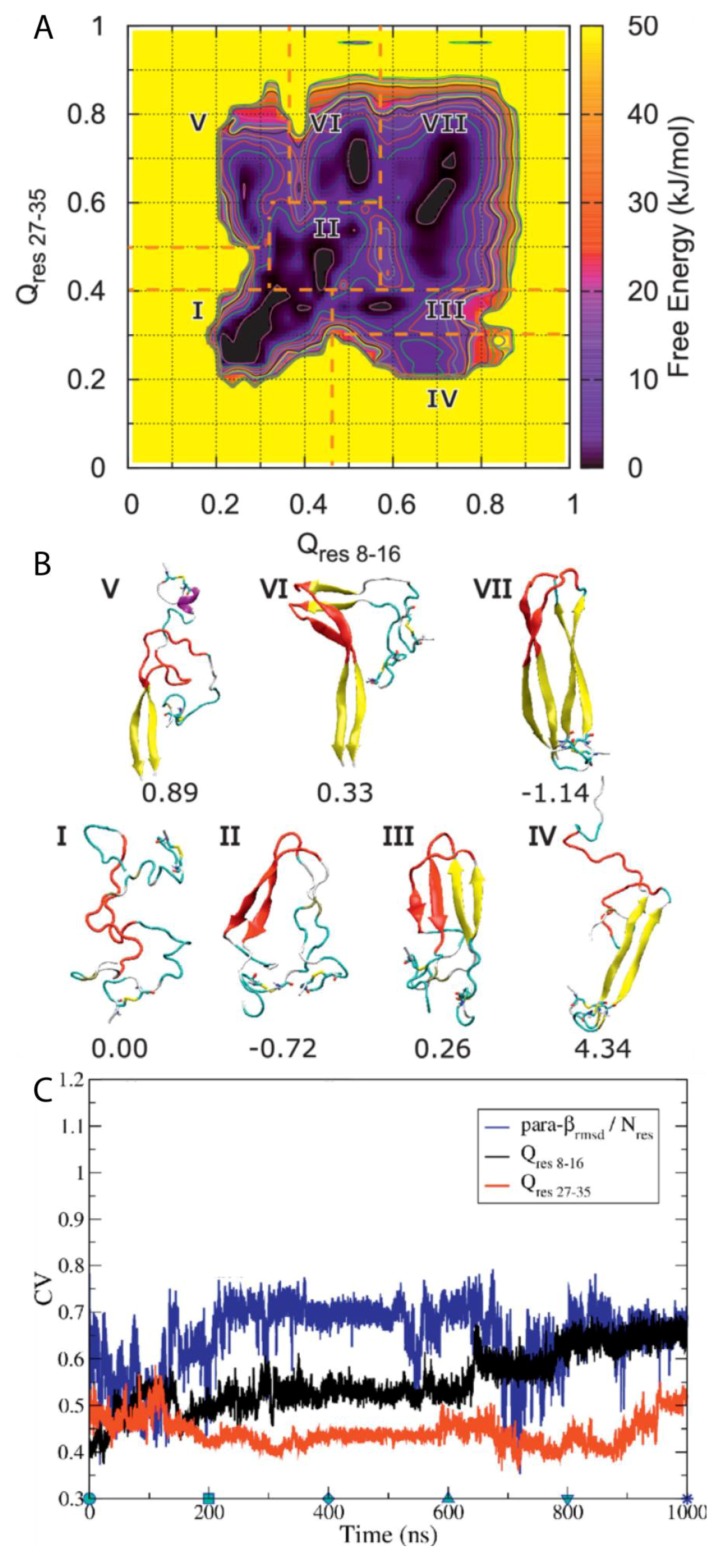
Characterisation of the dimerisation of hIAPP. (**A**) Progression of collective variables (CVs) with respect to simulation time. The inset is the free energy landscape of the system. (**B**). Representative structures from each free energy minimum. (**C**). Time evolution of Q_res 8–16_, Q_res 27–35_, and parallel β-RMSD. Adapted from Chiu and de Pablo [100]. Licensed under a Creative Commons Attribution (CC BY) license.

A subsequent study by Chiu and de Pablo used BEMD simulations to study the dimerisation of hIAPP using the Gromos96 53a6 force field with the SPC water model [100]. The authors aimed in particular to characterise the role in dimerisation of the core mutation region (residues 20–29), which, although known to be the amyloidogenic region for the process of aggregation, it is mostly unstructured in the mature fibril according to ssNMR data. Five CVs were used. The first two, Q_res 8–16_ and Q_res 27–35_ (CV1 and CV2), relate to the extent to which a conformation deviates from a U-shaped dimer, with β-sheets as the reference structure. The other three relate to ideal β-sheet structures in each monomer: parallel-β-RMSD for residues 8–16 (CV3), parallel β-RMSD for residues 27–35 (CV4), and parallel-β-RMSD for residues 20–29 (CV5). The resulting free energy surface was observed to contain seven regions, each corresponding to an intermediate state in a local free energy minimum, with a range of different conformations sampled during the simulation (Figure 15A). Two aggregation pathways were defined based on the representative structures of all the free energy minima and identified to be I→II→III→VII and I→II→VI→VII, with representative structures shown in Figure 15B. Intermediate I represents conformations with no secondary structure but with the monomers close to each other. Intermediate II contains a parallel β-sheet formed within residues 20–29, with residues 23–27 having a higher β-strand content [31]. In intermediate III, another set of β-sheets is observed. Intermediates IV and V exhibit a loss of β-sheet structure in the region 20–29. Intermediates VI and VII exhibit that the β-stranded region 20–29 is flanked by β-sheets, wherein the dimer in this last intermediate takes a fibril-like conformation. As shown in Figure 15C, during the first 100 ns the system moved from region II to region III, with both Q CVs increasing while parallel β-RMSD decreases, which indicates a reduction in the amount of transient β-sheet content in the core mutation region (residues 20–29). During the next 500 ns the dimer remained in region III and parallel-β-RMSD increased before adopting a constant value. Q_res8–16_ and Q_res27–35_ also remained nearly constant. These findings suggest that the core mutation region (residues 20–29) in the β-sheet structure may play a role in the stabilization of the dimer intermediate. At around 600 ns, Q_res8–16_ increases again, indicating that dimerisation continues during this period of time. During this time, it was also observed that parallel-β-RMSD_20–29_ decreased, which suggests the disruption of β-sheet structure. It was concluded that dimerisation is initiated by the formation and subsequent disruption of the core mutation region in β-sheets [31]. As shown in Figure 15C, the representative structures of each cluster reveal that the core mutation region transiently samples a loop, thus exploring the conformations that are also sampled by the monomeric form of hIAPP, as discussed previously. This region was indeed observed to be involved in the formation of transient β-sheets during dimerisation. In this study, the dimerisation of pramlintide was also characterised, and was observed to be mediated only by the N-and C-terminal regions. The core mutation regions of each monomer of pramlintide did not interact with each other, whilst other intermediate regions sampled conformations with higher free energy compared to hIAPP [100,101].

**Figure 16:**
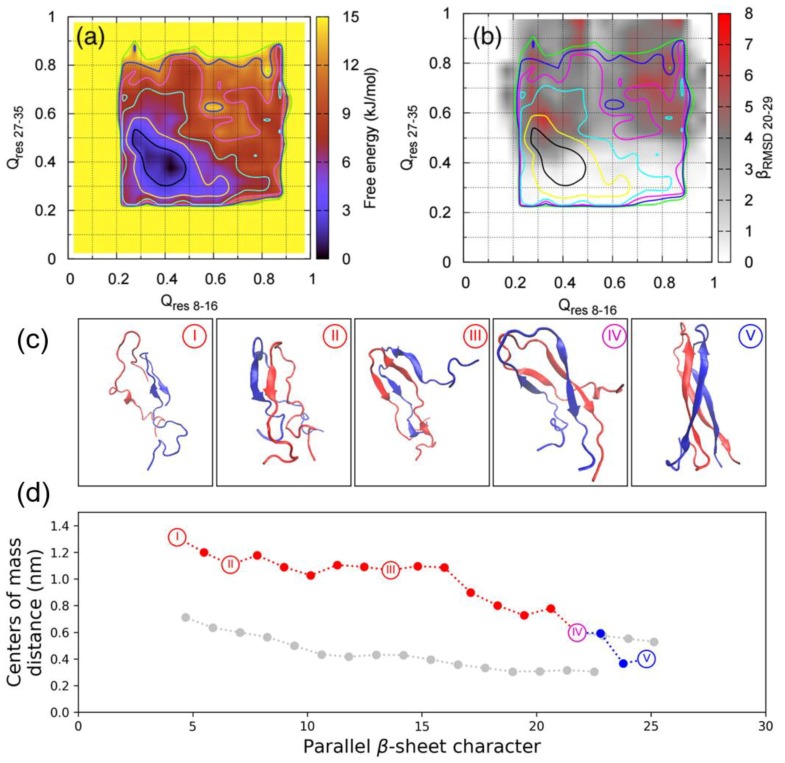
Characterisation of the dimerisation of hIAPP. (**a**) Free energy landscape of the hIAPP dimer as a function of Q_res 8–16_ and Q_res 27–35_. (**b**) Average amount of parallel β-sheet spanning residues 20–29 as a function of Q_res 8–16_ and Q_res 27–35_. (**c**) Representative structures of the conformational changes during dimerisation. (**d**) Dimerisation pathway obtained using the finite temperature string method. Adapted from Guo et al. [102] with copyright permissions from AIP Publishing.

A recent BEMD simulation study of the dimerisation of hIAPP by Guo et al. used the AMBERff99SB*-ILDN force field with the TIP3P water model [102]. This choice of force field was influenced by findings that the AMBERff99SB*-ILDN force field is more accurate in predicting the conformational landscape of hIAPP in solution, while GROMOS96 53a6 was seen to overstabilise the β-sheet structure [62]. Similar CVs were used: Q_res 8–16_, Q_res 27–35_, and parallel-β-RMSD_20–29_, along with the finite temperature string method to characterise the dimerisation pathway. The global minimum in the free energy corresponds to conformations that exhibit little similarity to the ideal U-shaped dimer. However, when the corresponding parallel-β-RMSD_20–29_ values are overlayed, the global minimum is separated into two regions: one with lower β-content, and another one with significantly higher β-content within the monomers (Figure 16a,b) [102]. The amount of parallel β-sheets formed between hIAPP monomers was also computed as a function of the distance between the centres of mass of the monomers. As the system progressed through the structures shown in Figure 16c, the closer distance between the monomers is accompanied by increases in parallel β-sheet content, with intermediate disordered dimeric conformations rearranging themselves. The process of dimerisation is predictably accompanied by an increase in protein–protein (between monomers) contacts; however, the number of protein–water contacts was predicted to fluctuate around a certain range during the entire process [102].

In an attempt to describe the formation of higher order aggregates, another study considered monomers, trimers, tetramers, and pentamers of hIAPP taken from the ssNMR structure of a fibril. A comparison was also made with rIAPP by substituting in all of the structures of those residues that are different in rIAPP. Conventional MD simulations were done using the CHARMM27 force field with the TIP3P water model. It was determined that as more monomers are added, the stability of each monomer (measured by the amount of secondary structure present) increased compared to that in the monomeric and dimeric forms, which have higher conformational flexibility. On the other hand, in the case of rIAPP there was a significant loss in stability as the aggregate increased in size from dimer to pentamer [103].

**Figure 17:**
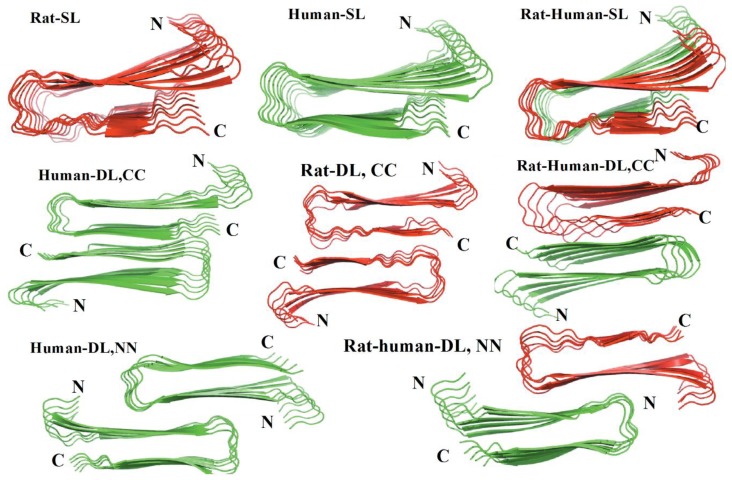
Single-layered (SL) and double-layered (DL) orientations of rIAPP and hIAPP homopentamers and heteropentamers, and the location of the water channel. These structures are arranged in different combinations of N-terminal and C-terminal order. Adapted from Berhanu and Hansmann [110]. Copyright 2014 Berhanu, Hansmann.

Another MD simulation study using the Amberff99SB force field with the TIP3P water model was conducted, with pentameric structures taken from fibrils of hIAPP to try to understand the interactions between single-layered and double-layered aggregates of hIAPP and rIAPP (Figure 17). The number of main chain and side chain hydrogen bonds between the β-sheets was larger in all of the different homopentamers of hIAPP: single layer (SL), double layer C-to-C-terminal (DL–CC), and double layer N-to-N-terminal (DL–NN). This was followed by heteropentamers, and finally the rIAPP homopentamers. The predicted free energies of binding were predicted to have the order human (DL–CC) > human (DL–NN) > rat–human (DL–CC) > rat–human (DL–NN) > human (SL) > rat–human (SL) > rat (DL–CC) > rat (SL). This was found to be related to the number of hydrogen bonds between β-sheets, which in turn is a function of the average distance between β-sheets. In rIAPP, the number of hydrogen bonds is significantly lower for both the main chain and the side chains due to the larger inter-sheet distances between hIAPP and rIAPP residues 23–27 (i.e., in the core amyloidogenic region). [104] The average distances between two β-sheets were also found to be smaller for the hIAPP C-to-C terminal interface model compared to rIAPP. These findings were explained on the basis that the C-terminal region acts as an anchor point for the β-hairpins between the two layers, limiting their conformational flexibility. The Pro residues in rIAPP increase the distance between β-sheets, making rIAPP aggregation less favourable compared to hIAPP. This is indeed reflected in lower free energies of binding in the rIAPP interface models compared to hIAPP. Interactions between hIAPP and rIAPP were observed through their common N-terminal region and β-sheet in residues 8–17. This was consistent with fourier transform infrared spectroscopy (FTIR) spectroscopy measurements that showed that residues 8–20 have characteristics of a typical β-sheet conformation [105]. The β-hairpin observed is similar to what has been observed in experimental and theoretical studies of hIAPP aggregates in membranes [106,107,108,109]. Finally, a water channel was observed within the β-hairpin structure (Figure 17) [110], which has been associated with the toxicity of hIAPP through pore formation leading to membrane leakage [111]. This water channel in the interior of the cavity of homo- and hetero-pentamers was observed to be formed by polar residues (Arg14, Ser28 (Pro28 in rIAPP), and Thr30), as observed in other simulation studies [106,112].

## 5. Cross-Aggregation of hIAPP with Aβ

It is believed that hIAPP has interactions at the molecular level with Aβ that may result in an increased risk of developing AD in patients suffering from T2D [18,113]. Deposits of hIAPP have been found in the temporal lobe of gray matter [114], leading to the hypothesis that hIAPP can cross-aggregate with Aβ in the pathogenesis of AD. Studies of the hetero-oligomerisation of hIAPP and Aβ have also been conducted in order to understand how these proteins interact and cross-aggregate. Hetero-oligomerisation of these proteins may involve an initial cross-seeding event, a process whereby one type of protein acts as a conformational template for another type of protein during aggregation.

Thioflavin-T (ThT) fluorescence assays have been used to study the aggregation kinetics between Aβ_42_ and hIAPP. In this assay ThT specifically binds to the β-sheet structure in a protein, resulting in the emission of fluorescence [115]. The aggregation kinetics of Aβ_42_ and hIAPP analysed after 30 h of incubation at a range of high concentrations (50, 25, and 12.5 µM) showed that the rates of aggregation followed the order Aβ_42_ > Aβ_42_ + hIAPP > hIAPP [115]. The authors suggested that cross-seeding is less efficient than homoseeding of pure Aβ_42_ but more efficient than homoseeding of hIAPP. In addition, the cross-seeded proteins formed less amyloid fibril than pure Aβ_42_ but more than hIAPP. An opposing trend was also observed where a longer lag phase for the initial aggregation stage was accompanied by an increased rate in the final stages of aggregation. This led to the speculation that in the beginning the solution comprises only monomers and some small oligomers. However, cross-aggregation cannot take place do to structural diversity and incompatibility because the homo-oligomers formed by both proteins have different orientations, unlike in a seed. Once a certain nucleation stage is achieved for each protein (i.e., homo-seeds), the proteins can begin to interact with each other. Furthermore, analysis of secondary structure using far-UV CD spectroscopy showed that during aggregation, pure hIAPP exhibits a transition from initial random coil to dominant α-helical conformation and some β-sheet content, consistent with previous findings (see Section 2.3), whereas pure Aβ_42_ initially samples random coil before increasing in α-helical and β-sheet content until it reaches a dominant β-sheet (50%) and α-helical (30%) structure. In the hetero-aggregates, however, both α-helical and β-sheet structures were initially observed, and which progressively converted to a dominant β-sheet structure. These observations suggest that the delay in the formation of β-sheet structure during hetero-oligomerisation occurs at the expense of the transformation of hIAPP from an α-helical to a β-sheet conformation [115]. Interestingly, a different study with Aβ_40_ showed that aggregation kinetics at a concentration of 32 µM after 15 h followed a different order: hIAPP > Aβ_40_ + hIAPP > Aβ_40_ [116].

**Figure 18:**
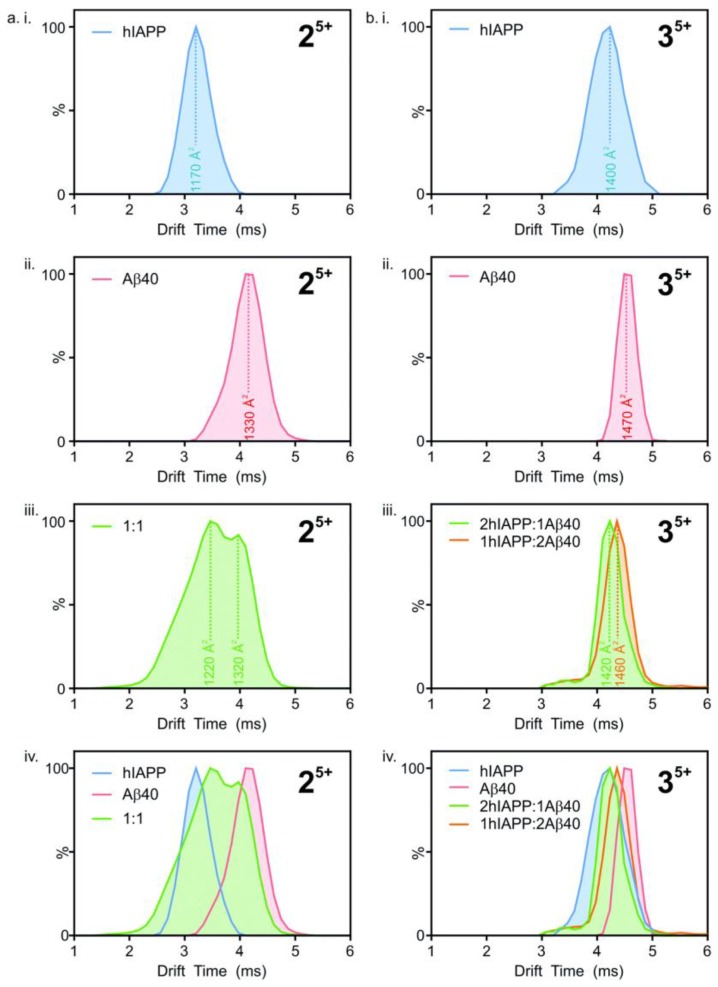
Collisional cross-section areas for dimers (**left**) and trimers (**right**) of Aβ and hIAPP at different molar ratios. Adapted from Young et al. [116]. Licensed under a Creative Commons Attribution (CC BY) license.

Ion mobility mass spectrometry has been used to study the formation of hetero-oligomers of hIAPP and Aβ_40_. Drift times (i.e., the time taken by ions to deflect from the sample and hit the sensor) were converted to collisional cross-section (CCS) areas, with the number of peaks representing the different conformations present (Figure 18). The heterodimeric system (Figure 18(aiii)), shows two peaks at different drift times, corresponding to two different conformations. However, the peaks fall between those seen for homodimers (Figure 18(aiv)). This suggests that the structure of the heterodimers is unique, although they are influenced by the structures of their respective constituents. Similarly, in the heterotrimeric system (Figure 18(biii,biv)), with ratios of 2:1 and 1:2 for hIAPP and Aβ_40_, respectively, the two conformations are different from each other as they have unique peaks that lie between the two homotrimeric peaks (Figure 18(bi,bii)), even if the difference between drift times is smaller compared to the dimeric system. The hetero-oligomers of hIAPP and Aβ_40_ thus have similar, but nonetheless unique conformations to those of their homo-oligomeric counterparts [116].

**Figure 19:**
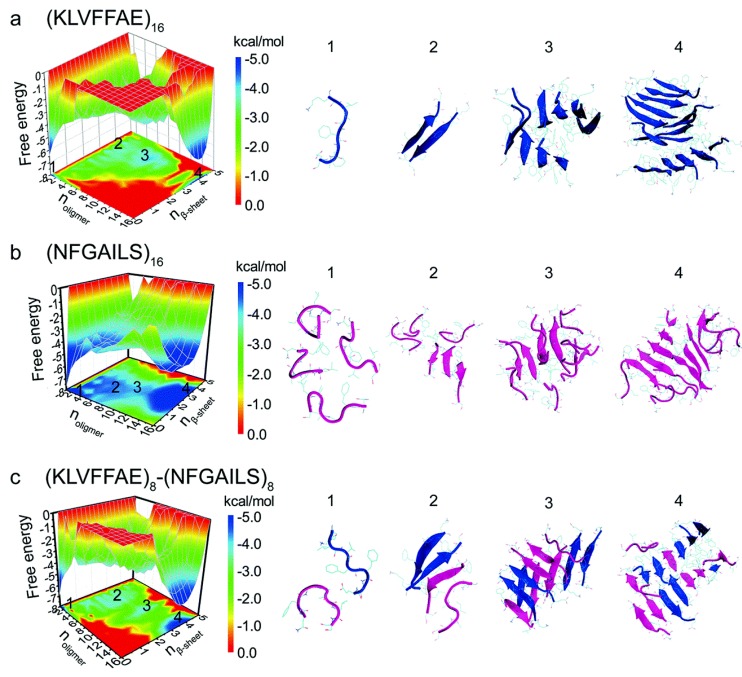
Free energy landscape and cluster representatives in each free energy minima: (**a**) Aβ_16–22_, (**b**) hIAPP_22–28_, and (**c**) Aβ_16–22_-hIAPP_22–28_. Adapted from Sun et al. [119]. Copyright 2013 American Chemical Society.

A study of cross- and self-aggregation using discrete molecular dynamics (DMD) simulations described the interactions between homo- and hetero-oligomers of two amyloidogenic fragments of hIAPP and Aβ. The first fragment is hIAPP_22–28_ (NFGAILS) containing an amyloidogenic sequence from the core mutation region which is capable of forming a cross β-sheet core in fibrils [117]. The second fragment is Aβ_16–22_ (KLVFFAE), which corresponds to one of the β-strands that constitute the core of Aβ_40_ fibrils [118]. Aggregation was studied with all systems in a 1:1 ratio. Fully extended peptides were used as a starting conformation with any pair-wise distance between them being no less than 1.5 nm. Due to its high hydrophobicity, Aβ_16–22_ exhibited a higher propensity to form β-sheets, even at the level of dimers. In the case of hIAPP_22–28_, formation of β-sheets upon aggregation was slower, which was reflected by the presence of more unstructured oligomers. Oligomers of hIAPP with less than six peptides exhibited random coil structures. When this number increased to eight or more peptides, there was a conformational transition from random coil to β-sheet. Potential of mean force calculations revealed how the free energy changed over a set of reaction coordinates: oligomer size, and the number of residues with β-sheet structure. The resulting free energy landscape revealed that Aβ_16–22_ homo-oligomers have the lowest values of the free energy, whereas hIAPP_22–28_ homo-oligomers have higher values, indicating that the former are more stable (Figure 19a,b). In the case of cross-aggregation, the values of the free energy were observed to be lower than those of hIAPP_22-–28_ homo-oligomers and similar to those of Aβ_16–22_ homo-oligomers [119].

In another study, ThT fluorescence assays were combined with DMD simulation followed by replica exchange DMD (REXDMD) simulations to study the self- and cross-aggregation between Aβ_42_ and hIAPP in dimeric systems. The structures of Aβ_42_ obtained by solution NMR in an organic solvent [120] and hIAPP obtained by solution NMR in a micellar environment [53] were used to commence the simulations. Characterisation of the aggregation kinetics with an equimolar ratio of both peptides at different concentrations (3–5 µM) showed that the rates of aggregation were hIAPP > hIAPP + Aβ_42_ > Aβ_42_ [121,122], which is inconsistent with the above-described study conducted at significantly higher concentrations. [115,116] Analysis of atomic contacts showed that Aβ_42_-hIAPP hetero-oligomers had a larger number of contacts between residues compared to the corresponding homo-oligomers, with hIAPP homo-oligomers showing the smallest number of contacts of all. This suggests that cross-species interactions dominate self-aggregation. The presence of opposite electric charges in the two proteins (Aβ_42_: −3 and hIAPP: +2) increases cross aggregation between the proteins. Interaction hotspots with high-binding probability were identified in both self- and cross-aggregation systems. In Aβ_42_, these residues were 19–22, 27–32, and 35–40, while in hIAPP, these residues were 8–18 and 22–28 [123]. Formation of a heterodimer was marked by unfolding of the Aβ_16–22_ segment, i.e., the central hydrophobic core, which suggests that the binding of hIAPP to Aβ_42_ reduces the free energy barrier of the unfolding of the helical region 16–22 of Aβ_42_, hence promoting the cross-aggregation with a reduced lag phase time for nucleation [124].

The key residues in hIAPP involved in its cross-aggregation with Aβ_40_ were determined using alanine scanning, a range of biophysical methods, and MD simulations [125]. cMD simulations were performed using the AMBER ff14SB force field with the TIP3P water model on hexamers of HIAPP whose structure was determined by X-ray crystallography of amyloidogenic segments (NNFGAIL) of hIAPP [44]. Substitutions were made to understand the role of segments in the sequence, as well as individual residues of hIAPP. Aromatic/hydrophobic residues Phe23 and Ile26, which are part of the core mutation region (i.e., FGAIL) are known to act as hot spots for its interaction with Aβ_40_ but not for self-aggregation [44,117]. In hIAPP, individual substitution of residues Phe15, Leu16, Phe23, and Ile26 by Ala resulted in a strong reduction in binding affinity towards Aβ40. Finally, residues Phe15, Leu16, Phe23, and Ile26 in the amyloidogenic core of hIAPP (region 15–29) were determined to act concertedly to enable self- and cross-aggregation with Aβ40/42. 

A MD simulation study using the CHARMM27 force field and the TIP3P water model aimed to provide a topological characterisation of the cross-interactions between hIAPP and Aβ42 [126]. The fibril structures of hIAPP [44] and Aβ42 [40] were used to construct homo-hexamers and various hetero-dodecamers. Conformations taken at the end of the simulations were used to compute the conformational free energies upon energy minimisation and Monte Carlo simulations. Polymorphic single layer conformations of Aβ42-hIAPP with parallel and anti-parallel arrangements were determined to have the highest population compared to other single layer and all double layer conformations. The parallel and anti-parallel arrangements of these single layer conformations were predicted to be energetically favoured, suggesting a strong tendency for cross-seeding between Aβ42 and hIAPP [126].

**Figure 20:**
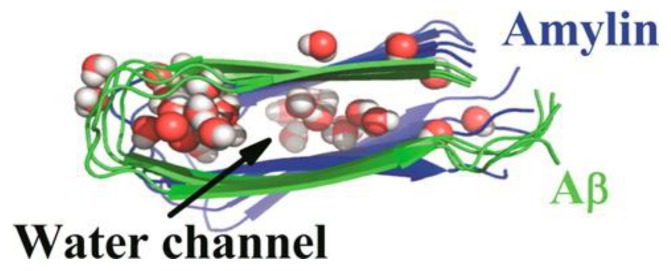
Location of the water channel present in the hetero-oligomer of Aβ_15–40_ and hIAPP_10–35_. Adapted from Berhanu and Hansmann [106]. Copyright 2015 American Chemical Society.

Another MD simulation study using the Amber ff99SB force field with the TIP3P water model aimed to characterise octamers of hIAPP, Aβ, and hIAPP-Aβ. These structures were constructed from the ssNMR structures of the fibrils of hIAPP and Aβ but were truncated on the basis that the N-terminal regions of these proteins are disordered, such that only the Aβ15-40 and hIAPP10-35 regions were retained [44,127]. All three octamers were observed to be stabilized by main chain hydrogen bonds between monomers. Hydrophobic interactions were seen to play an important role in retaining the “U”-shape of the oligomers by forming inter-peptide interactions between non-polar residues in the L13ANFL17 and A25ILSS29 regions of hIAPP, and the L17VFFA21 and A30IIGL34 regions of Aβ. In all of the oligomers, the core β-strand-turn-β-turn motif was stabilized by face-to-face hydrophobic interactions. The distance between two β-sheets in the hIAPP octamers was observed to be smaller than at the interface between Aβ and IAPP in the cross-seeded octamer, indicating compensation by hIAPP to stabilize the interaction. The inter-chain salt bridge (involving residues Asp23 and Lys28) present between the loop/U-turn connecting two β-sheets in the Aβ and Aβ-hIAPP octamers was observed to stabilize a hydration cavity, but not in the hIAPP octamer, due to the absence of the salt bridge. A water channel was observed in the β-strand-turn-β-strand motif of the three systems; however, owing to the nature of the residues present, the location of the water channel was different. In Aβ, the water channel was found in the loop region near a salt bridge, whilst in hIAPP, it was found between the β-strands, because the loop region is rich in hydrophobic residues. The average number of water molecules in the channel had the order Aβ > Aβ-hIAPP > hIAPP. These water channels in amyloids are believed to be associated with water leakage through cell membranes as a mechanism of their toxicity (Figure 20), as has been reported in in vitro studies [128,129].The predicted free energy of binding showed that the interactions within the monomers were favourable [106].

**Figure 21:**
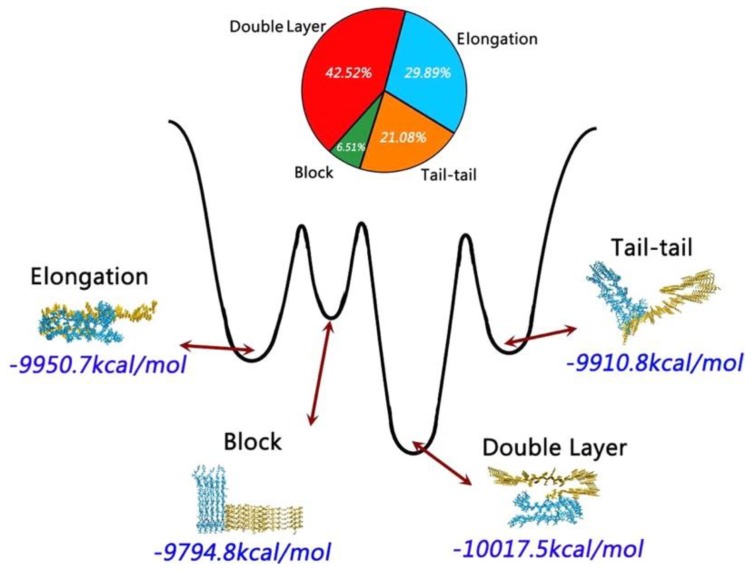
Structural ensemble of hetero-pentamers of Aβ_42_-hIAPP, showing their relative proportion and free energies for their mutual orientations. Adapted from Zhang et al. [131]. Copyright 2015 American Chemical Society.

A coarse-grained (CG)-based REMD simulation study using the Martini force field followed by all-atom simulations using the CHARMM27 force field with the CMAP correction and the SPC/E water model characterised the cross-seeding between the pentamers of Aβ_42_ and hIAPP, whose structure were taken from fibrils [40,130]. These pentameric structures were determined to interact in various preferred orientations: double-layer model (43%), elongation model (30%), tail-tail model (21%) and block model (7%) (Figure 21) [131].

With the exception of the studies of dimerization discussed above, all other MD simulations of the formation of hetero-oligomers of hIAPP and Aβ have been carried out using structures derived from the corresponding fibril structures of each protein, thereby introducing a significant structural bias. This approach has prevented the investigation of the actual mechanism of formation of hetero-oligomers from unstructured monomers and smaller-sized oligomers.

## 6. Conclusions

Characterisation of the structure and aggregation of hIAPP is of the utmost importance due to its association with T2D, as well as its ability to cross-aggregate with other amyloidogenic proteins such as Aβ, which may in turn increase the likelihood of AD. However, the study of the structure and interactions of hIAPP in solution at physiological conditions is hampered by its intrinsically disordered nature and its large tendency for aggregation. Experimental methods such as XRD, NMR, CD, EPR, IR, and mass spectrometry have all been used to characterise the monomeric form of hIAPP; however, no method is suitable for use at physiological conditions due to the resulting fast aggregation of this protein. Nonetheless, some approaches to prevent aggregation have been used, such as low pH and low temperature, or the use of detergent micelles to mimic the cell membrane environment. The structural properties of hIAPP determined in those conditions have reached similar findings, showing that hIAPP (and rIAPP) transiently sample α-helical structures, with the C-terminal region of rIAPP being more disordered due to the presence of three Pro substitutions. Understanding the structural properties of the monomer of hIAPP in physiological conditions remains the first step in establishing the molecular mechanism of aggregation of this protein. It is also equally important to understand how amyloidogenesis initiates, and the conformational changes and interactions that occur as aggregation proceeds.

Multiple MD simulation approaches have been used to characterise the monomeric structure of hIAPP and rIAPP in solution. However, MD simulations can have limitations due to the choice of force field, starting structure and enhanced sampling method. Some force fields have been shown to introduce bias in the simulations towards largely folded compact structures, whilst others predict the lowest conformational energy state to be a complete random coil. The starting structure (e.g., random coil or α-helical structures) has also been seen to influence the predictions of simulations even when enhanced sampling methods have been used to increase sampling efficiency. Enhanced sampling methods such as BEMD, REST2, HT-REMD, and T-REMD have been tested for their ability to characterise the monomeric structure of hIAPP. Most methods have come to the same conclusion, indicating that the most stable conformation of hIAPP corresponds to a random coil, with some tendency to adopt some α-helical and β-sheet conformations. 

Various computational approaches have been used to characterise the formation and structure of homo-aggregates of hIAPP. The formation of dimers of hIAPP has been determined to involve the interaction between the core mutation regions of two monomers. The core mutation region (residues 20–29), and especially residues Arg22 and Phe23, have been shown to be essential for the interaction with additional monomers of hIAPP. By contrast, the residues within this same range in rIAPP are different, giving rise to its non-amyloidogenic nature. Dimerisation has been proposed to involve the conversion of hIAPP from a predominantly α-helix/random coil structure in the monomer to a dimer comprised predominantly of α-helix/β-sheet structure. As dimerisation proceeds, interacting monomers sample multiple non-fibrillar states before ultimately adopting a fibril-like conformation. In particular, the core mutation region has been predicted to assume transient β-sheet structure and random coil conformations on the path towards forming a stable dimer. Interestingly, this region has been characterised to be a disordered loop in the NMR structures of fibrils of hIAPP, thus suggesting that further structural rearrangement occurs during later stages of fibrillation. On the other hand, the overall the stability of higher-order aggregates is predicted to increase with the addition of more monomeric units. Understanding the process of aggregation and specifically the core mutation region may be useful for the development of small-molecule inhibitors of aggregation. Pramlintide, a version of hIAPP modified to contain the three prolines found in rIAPP, has been used as a drug.

MD simulation studies of the cross-aggregation between hIAPP and Aβ_42_ have revealed the presence of specific interacting hot spot regions between the two proteins: residues 19–22, 27–32, and 35–40 in Aβ_42_, and 8–18 and 22–28 in hIAPP. The interaction of these two proteins has been predicted to facilitate the gradual conversion of the transient conformations of hIAPP to β-sheet structures, accelerating aggregation. The conformers of hetero-oligomers were found to be topographically different from those observed in the respective homo-oligomers. Formation of hetero-oligomers was observed to be accompanied by a more favourable change in free energy than in the formation of homo-oligomers of hIAPP, but similar to that of the formation of homo-oligomers of Aβ_42_. Formation of hetero-oligomers from pre-formed homo-oligomers of each species was predicted to involve unique relative orientations between the homo-oligomers, such as the double-layer model, elongation model, tail-tail model, and block model. A water channel has also been observed in hetero-oligomeric fibrils, which are believed to be associated with the cell membrane toxicity of hIAPP. 

Most of the MD simulation studies of aggregation have relied on the use of pre-formed structures of homo-oligomers of hIAPP and Aβ_42_ to investigate hetero-oligomerisation. This approach inevitably introduces an initial structural bias into the simulations, and prevents the characterisation of the gradual changes in conformation that are likely to occur as disordered monomers of hIAPP and Aβ_42_ self- and cross-interact to form higher-order disordered hetero-oligomers. Future studies are needed to characterise these early stages of self- and cross-aggregation in detail. 

## Figures and Tables

**Table 1:** Sequence of islet amyloid polypeptides. The core mutation region is highlighted in blue, with cysteine residues forming a disulfide bond are linked together by brackets, and the differences between the human (hIAPP) and rat (rIAPP) forms are highlighted in red.

hIAPP-K(CNTATC)ATQRLANFLVHSSNNFGAILSSTNVGSNTY-NH2
rIAPP-K(CNTATC)ATQRLANFLVRSSNNLGPVLPPTNVGSNTY-NH2
